# Hematopoietic pannexin 1 function is critical for neuropathic pain

**DOI:** 10.1038/srep42550

**Published:** 2017-02-14

**Authors:** Janelle L. Weaver, Sanja Arandjelovic, Gregory Brown, Suresh K. Mendu, Michael S. Schappe, Monica W. Buckley, Yu-Hsin Chiu, Shaofang Shu, Jin K. Kim, Joyce Chung, Julia Krupa, Vesna Jevtovic-Todorovic, Bimal N. Desai, Kodi S. Ravichandran, Douglas A. Bayliss

**Affiliations:** 1 Department of Pharmacology, University of Virginia, Charlottesville, Virginia 22908, USA; 2 Department of Microbiology, Immunology, Cancer Biology, University of Virginia, Charlottesville, Virginia 22908, USA; 3 Carter Immunology Center, University of Virginia, Charlottesville, Virginia 22908, USA; 4 Center for Cell Clearance, University of Virginia, Charlottesville, Virginia 22908, USA; 5 Department of Anesthesiology, University of Colorado, Aurora, Colorado 80045 USA

## Abstract

Neuropathic pain symptoms respond poorly to available therapeutics, with most treated patients reporting unrelieved pain and significant impairment in daily life. Here, we show that Pannexin 1 (Panx1) in hematopoietic cells is required for pain-like responses following nerve injury in mice, and a potential therapeutic target. Panx1 knockout mice (*Panx1*^−/−^) were protected from hypersensitivity in two sciatic nerve injury models. Bone marrow transplantation studies show that expression of functional Panx1 in hematopoietic cells is necessary for mechanical hypersensitivity following nerve injury. Reconstitution of irradiated *Panx1* knockout mice with hematopoietic *Panx1*^−/−^ cells engineered to re-express Panx1 was sufficient to recover hypersensitivity after nerve injury; this rescue required expression of a Panx1 variant that can be activated by G protein-coupled receptors (GPCRs). Finally, chemically distinct Panx1 inhibitors blocked development of nerve injury-induced hypersensitivity and partially relieved this hypersensitivity after it was established. These studies indicate that Panx1 expressed in immune cells is critical for pain-like effects following nerve injury in mice, perhaps via a GPCR-mediated activation mechanism, and suggest that inhibition of Panx1 may be useful in treating neuropathic pain.

Neuropathic pain is increasingly recognized as a major clinical and societal problem, contributing to the almost $635 billion spent annually on chronic pain in the US^1^,^2^. Unlike acute pain, which is protective, neuropathic pain is a chronic, burning, “pins and needles” sensation that does not respond well to currently available therapeutics. The refractory nature of this condition negatively impacts patient quality-of-life, leading to steep economic costs associated directly with the condition and indirectly via lost productivity and the use of opiate drugs as a primary treatment option (e.g., drug addiction and associated crime). A better understanding of the molecular and cellular bases for pathogenesis of neuropathic pain may yield more effective therapeutics.

Neuropathic pain results from physicochemical insults to the nervous system, which trigger a chronic inflammatory response and subsequent functional remodeling of sensory circuits that underlie the persistent pain and/or sensory hypersensitivity^1^,^3^. Different immune cell types have been implicated in the pathological process at multiple peripheral and central sites, with complex intercellular signaling among and between immune and neural cells orchestrated by various inflammatory mediators (e.g., ATP/UTP, cytokines, BDNF, receptor agonists)^1^,^3^,^4^,^5^. A role for the Pannexin 1 (Panx1) membrane channel in neuropathic pain has been advocated because it is widely-expressed (in neurons, glia and immune cells)^6^,^7^, and because Panx1 can be activated by various mechanisms that are relevant to nerve injury^1^,^3^. For example, Panx1 may be activated by inflammatory mediators (e.g., TNFα^8^), G protein-coupled receptors (GPCRs: e.g., P2Y6^9^), apoptotic signaling (e.g., caspase cleavage^10^), or coupling to ionotropic receptors (P2X7, NMDA^6^,^11^). When the channel is activated, the release of ATP and other inflammatory metabolites via Panx1 could, in principle, recruit immune cells and drive inflammation and pain^1^,^3^,^12^. Indeed, application of non-selective pharmacological tools has uncovered a relatively undefined function of Panx1 in neuropathic pain^11^,^13^,^14^,^15^. However, in the absence of specific genetic tools, a functional role for other protein targets of non-selective Panx1 inhibitors cannot be ruled out. Furthermore, Panx1 contributions have been attributed primarily to its expression in spinal cord or primary sensory neurons^13^,^14^,^15^, with proposed mechanisms of channel activation focusing on ionotropic receptor coupling to Panx1^13^,^14^,^16^; there remains only scant evidence for this prevailing model.

Here, we used global Panx1 knockout mice (*Panx1*^−/−^) and two sciatic nerve injury models of trauma-induced neuropathy to provide genetic evidence for a major role of Panx1 in neuropathic pain. Strikingly, the mechanical hypersensitivity that typically develops after nerve injury was fully abrogated in *Panx1*^−/−^ mice in both models. Surprisingly, this persistent pain-like response following nerve injury required Panx1 expression in circulating hematopoietic cells – not in neurons or astrocytes. Mechanical hypersensitivity following nerve injury was restored in *Panx1*^−/−^ mice that received bone marrow transplantation either with cells from *Panx1*^+/+^ mice or with cells from *Panx1*^−/−^ mice retrovirally-transduced with wild-type Panx1. Notably, this latter rescue was not observed with re-expression of Panx1 channels containing a point mutation rendering them resistant to activation by GPCRs, suggesting a GPCR-mediated channel activation process. From a therapeutic standpoint, mice treated with two chemically-distinct Panx1 blockers were protected from mechanical hypersensitivity in this neuropathic pain model. These data demonstrate that Panx1 expression in hematopoietic cells is both necessary and sufficient for its effects on nerve injury-induced pain, and that pharmacological inhibition of Panx1 could be a viable therapeutic alternative for the prevention and/or treatment of neuropathic pain.

## Results

### Panx1^−/−^ mice are protected from the hypersensitivity that typically accompanies nerve injury

To assess whether Panx1 contributes to neuropathic pain, we obtained global Panx1 knockout mice (*Panx1*^−/−^) that were maintained on a C57BL/6 background (Fig. S1A–C)^17^. *Panx1*^−/−^ mice had no gross abnormalities or other apparent differences from wild-type littermates (*Panx1*^+/+^ mice), as previously reported for other lines of Panx1 knockout mice^6^, and heterozygous breedings yielded the expected Mendelian distribution of genotypes. *Panx1*^−/−^ mice performed equally to wild-type mice in sensorimotor tests (Fig. S1D), and importantly, did not demonstrate any significant differences in normal (uninjured) sensitivity to heat or mechanical stimuli (Fig. S1E,F), ruling out systemic defects in nociception; for these and all studies reported herein, experiments and image analysis were performed by investigators blinded to genotype and/or treatment.

**Figure 1:**
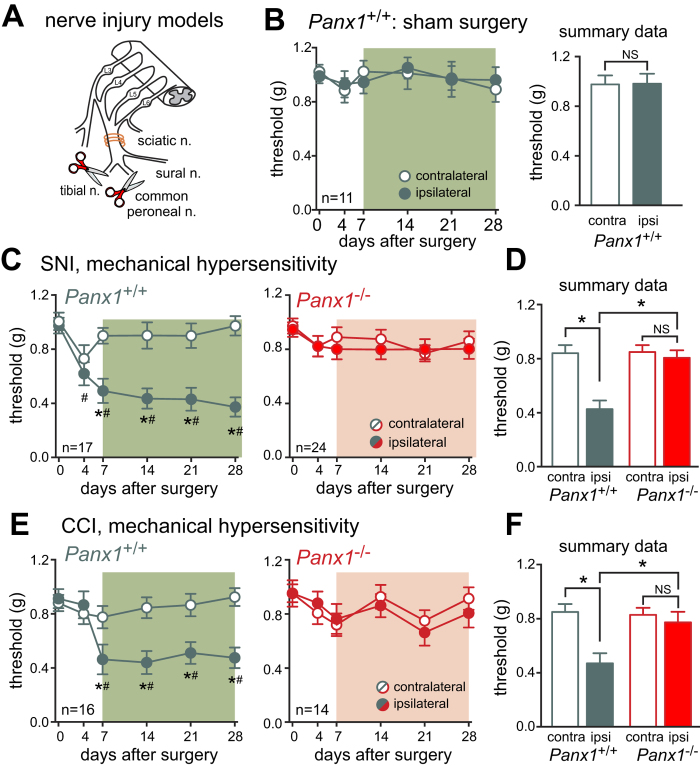
*Panx1*^−/−^ mice are resistant to SNI-induced mechanical hypersensitivity. (**A**) SNI and CCI models. (Image adapted from^55^) (**B**) Absence of mechanical hypersensitivity in *Panx1*^+/+^ mice undergoing sham surgery (n.s., p > 0.46 by two-way RM ANOVA). (**C**) Wild-type littermates underwent unilateral SNI and developed mechanical hypersensitivity in the operated limb. In contrast, *Panx1*^−/−^ mice were protected from mechanical hypersensitivity. (**D**) Summary data (for **C**) is the mean (±SEM) of the threshold value for each mouse, averaged over the period from POD7-28 (depicted by the shaded region in the time series). (**E,F**) Like SNI, mechanical hypersensitivity following CCI is completely absent in *Panx1*^−/−^ mice while wild-type littermates experience robust hypersensitivity. In this and all other figures showing the time course of mechanical or thermal sensitivity, two-way RM ANOVA was used for statistical analysis, with Tukey’s test for pairwise comparisons: ^#^p < 0.05 versus day 0; *p < 0.05 for contralateral versus ipsilateral paw. For all figures showing summary data, the averaged value from each mouse from POD7-28 was treated as a single data point in calculating group means that were compared by two-way RM ANOVA; *p < 0.05 between indicated groups, with Sidak’s test used for pairwise comparisons.

Hypersensitivity to sensory stimuli (or allodynia) is a common feature of neuropathic pain, with patients mainly complaining of increased sensitivity to touch^1^. To induce mechanical allodynia indicative of neuropathic pain, we performed a unilateral spared nerve injury (SNI)^18^ on male and female wild-type (*Panx1*^+/+^) and Panx1-deleted mice (*Panx1*^−/−^) and assessed mechanical sensitivity over the next 28 days (Fig. 1A). As expected, *Panx1*^+/+^ mice developed robust and persistent mechanical hypersensitivity ipsilateral to the injured sciatic nerve beginning between 4 to 7 days and continuing until at least 28 days after surgery, as evidenced by a decrease in the threshold for response to stimulation by von Frey filaments (Fig. 1C,D). In marked contrast to wild-type mice, *Panx1*^−/−^ mice were completely protected over the entire 28 day testing period (Fig. 1C,D). Mechanical sensitivity was unchanged in the paw contralateral to the nerve injury for either genotype; moreover, sham-operated mice exhibited no change in mechanical hypersensitivity in either paw over an equivalent time period (Fig. 1B). Sex-dependent differences in mechanisms of neuropathic pain have been reported^5^, but these do not appear to apply to Panx1 contributions since we observed equivalent Panx1-dependent effects on ipsilateral mechanical sensitivity following SNI in both male and female mice (Fig. S2A,B). Thus, Panx1-deleted mice, male and female, are resistant to SNI-induced mechanical allodynia.

We used a second traumatic injury model to confirm these findings. The sciatic nerve chronic constrictive injury (CCI) model (Fig. 1A) can be associated with greater variability^18^, but it has the advantage of provoking both mechanical and heat hypersensitivity^19^. Indeed, following CCI surgery, *Panx1*^+/+^ mice developed persistent mechanical (Fig. 1E,F) and heat hypersensitivity (Fig. S2C,D). In contrast, *Panx1*^−/−^ did not develop mechanical hypersensitivity (Fig. 1E,F), and exhibited only mild hypersensitivity to heat (Fig. S2C,D). The degree of injury resulting from CCI was similar between experimental groups, since there were no differences in the number of preserved axons or degenerating profiles in semithin sections distal to the site of nerve injury from *Panx1*^+/+^ and *Panx1*^−/−^ mice (Fig. S2E,F).

Thus, hypersensitivity in two different models of traumatic nerve injury was significantly attenuated by Panx1 deletion. We focused on the more robust SNI model for subsequent experiments.

### Panx1 expression in bone marrow cells mediates neuropathic pain

We first tested the prevailing model that Panx1 expression in either neuronal or glial cells accounts for its contributions to neuropathic pain. To this end, we crossed mice with “floxed” Panx1 alleles (*Panx1*^*fl/fl*^) to specific Cre recombinase mouse lines for gene deletion in neurons and astrocytes, i.e., to Syn-Cre and GFAP-Cre mice, respectively. Neuronal Cre expression is widespread in Syn-Cre line, including peripheral sensory neurons^20^, while in the GFAP line, Cre expression is primarily, but not exclusively observed in astrocytes (i.e., it is also expressed in some neural progenitors and oligodendrocytes)^21^,^22^. Surprisingly, and despite demonstrable knockdown of Panx1 in semi-purified neuron and astrocyte preparations, the mechanical hypersensitivity induced by SNI was fully retained in both sets of conditional knockouts (Fig. S3). This suggested that the major contribution of Panx1 in this model of neuropathic pain is not likely due to its expression in neurons or astrocytes, and we re-directed our attention to cells of the immune system.

**Figure 2:**
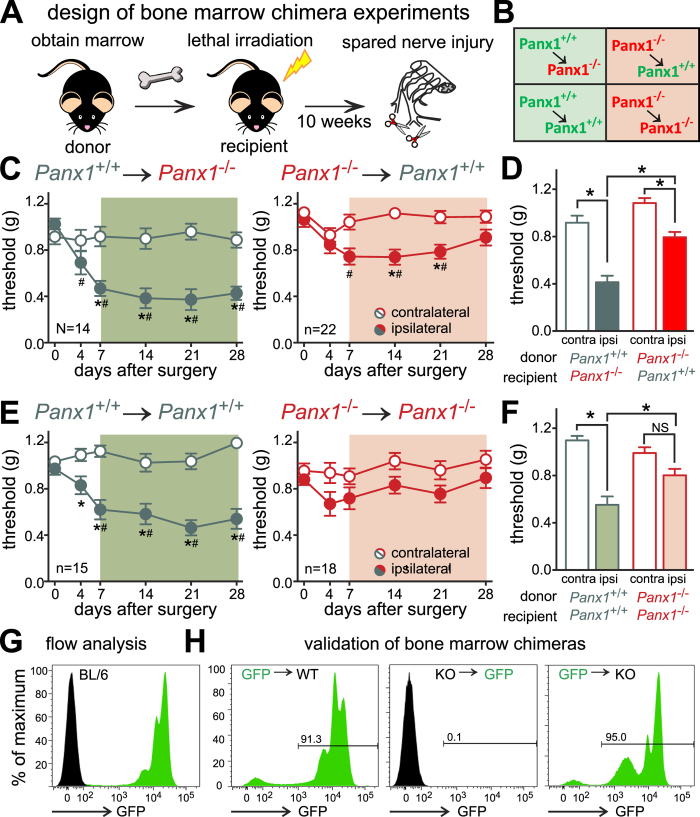
Bone marrow transplantation reveals a critical role for Panx1 in hematopoietic cells following nerve injury. (**A**,**B**) Study design^55^. (**C,D**) Mechanical hypersensitivity following SNI was restored in *Panx1*^−/−^ mice reconstituted with wild-type marrow and diminished in *Panx1*^+/+^ mice reconstituted with *Panx1*^−/−^ marrow. (**E,F**) SNI-induced mechanical hypersensitivity in mice reconstituted with cells of the same genotype. (**G**) Flow cytometry showing GFP fluorescence in bone marrow from C57BL/6 mice or UBI-GFP mice. (**H**) GFP fluorescence after bone marrow transplantation of cells from UBI-GFP or mice of the indicated Panx1 genotypes demonstrating successful irradiation and replacement. Statistical analysis of behavioral data as in Fig. 1.

Panx1 is expressed in many types of immune cell^6^,^12^, and inflammatory purinergic mechanisms have been implicated in chronic neuropathic pain^3^,^23^,^24^. We therefore performed bone marrow transplantation studies to test the contribution of Panx1 in bone marrow cells to nerve injury-induced mechanical allodynia, according to the illustrated protocol (Fig. 2A,B). There was no effect of the transplantation and reconstitution procedure on mechanical sensitivity since paw withdrawal threshold values were not different among any of the transplanted groups prior to nerve injury (see day 0 in Fig. 2C,E, which was 10 weeks after transplantation; P = 0.128 by ANOVA), and were also similar to those of naïve *Panx1*^+/+^ and *Panx1*^−/−^ mice (*cf.* day 0 in Fig. 1C,E). Strikingly, when *Panx1*^−/−^ recipient mice were reconstituted with *Panx1*^+/+^ cells from wild-type mice, the hypersensitivity following SNI was fully restored (Fig. 2C,D). Conversely, in wild-type mice reconstituted with *Panx1*^−/−^ cells, the hypersensitivity following injury was significantly blunted, and it resolved fully by the end of the test period (at 28 days post-injury, Fig. 2C,D). Importantly, after bone marrow transplantation between donors and recipients of the same genotype there was no difference in mechanical sensitivity after nerve injury, by comparison to the relevant non-transplanted groups (*cf.* Fig. 2E,F with Fig. 1C,D). In a subset of mice used for behavioral experiments (n = 2–3 mice/group), we confirmed that cells in the circulation (not shown) and the bone marrow (Fig. 2G,H) of transplanted mice were of donor origin by flow cytometry using mice that ubiquitously express green fluorescent protein (UBI-GFP mice). These data indicate that expression of Panx1 in bone marrow-derived cells is sufficient for its contribution to this model of neuropathic pain.

**Figure 3:**
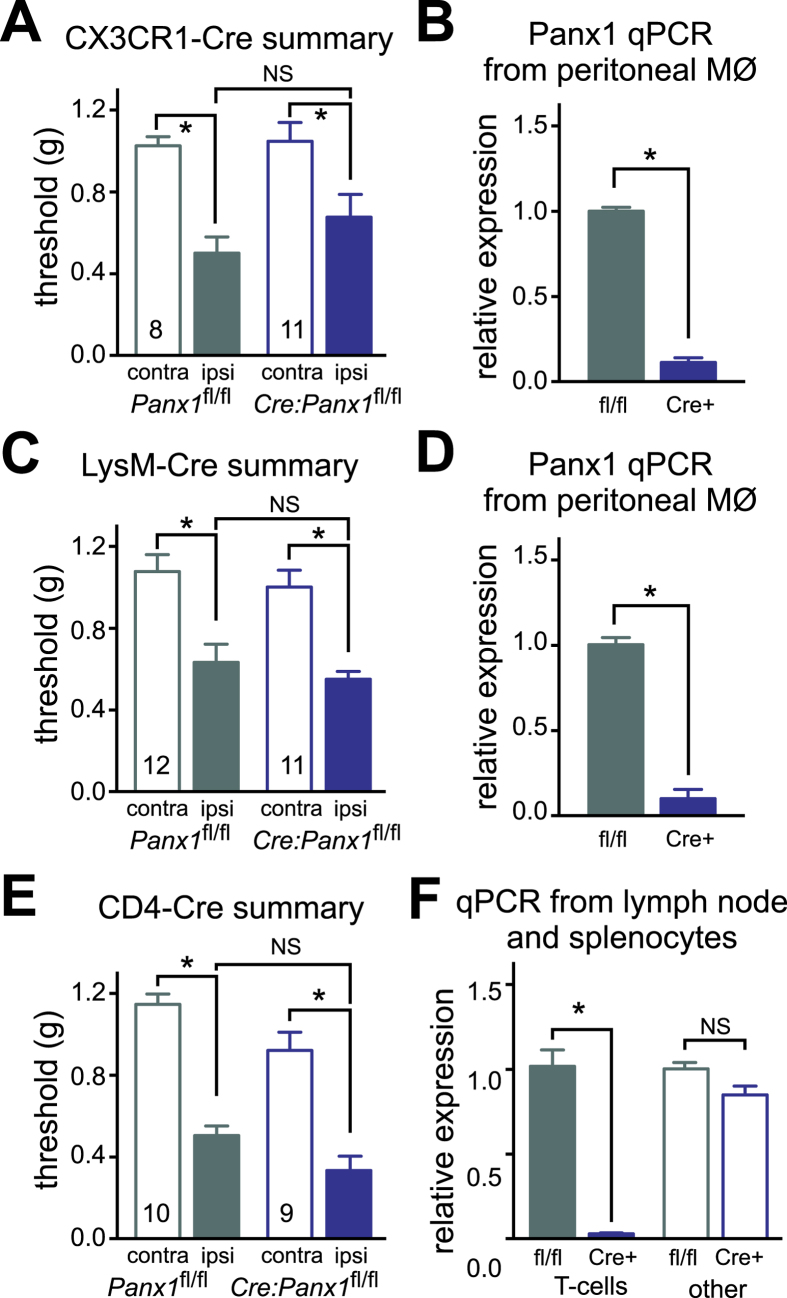
Removal of Panx1 from macrophages, myeloid cells or T cells does not prevent pain. (**A,C,E**) Summary data of effects of SNI on mechanical threshold in *Panx1* ^*fl/fl*^ mice and CX3CR1-Cre littermates (**A**), LysM-Cre littermates (**C**), or CD4-Cre littermates (**E**). (**B,D,F**) RT-qPCR for Panx1 (relative to HPRT) from peritoneal macrophages (**B**, n = 3–7, *p < 0.05, unpaired two-tailed t-test), from peritoneal macrophages (**D**, n = 5, *p < 0.05, unpaired two-tailed t-test) and from acutely isolated T cells (**F**, n = 4–5, *p < 0.05, one-way ANOVA followed by Tukey’s multiple comparisons test). See Fig. S4 for time course of changes in mechanical hypersensitivity.

In consideration of our results from the bone marrow transplants, and given the importance of macrophages/microglia in response to nerve injury^3^,^4^,^24^, we crossed *Panx1*^*fl/fl*^ mice to two different macrophage/myeloid-specific Cre lines: CX3CR1-Cre and LysM-Cre. These two Cre lines differ in the extent and subtype of cells targeted: strong Cre expression is seen in macrophages and microglia with both CX3CR1-Cre and LysM-Cre, but CX3CR1-Cre is expressed in a greater percentage of microglia^25^ and LysM-Cre is expressed more broadly in myeloid cells, including granulocytes^26^. Despite the readily observed loss of Panx1 in macrophages from both CX3CR1-Cre/*Panx1*^*fl/fl*^ and LysM-Cre/*Panx1*^*fl/fl*^ mice (Fig. 3B,D), they displayed a nerve injury-induced pain-like phenotype that was indistinguishable from floxed Cre(−) littermates (Figs. 3A,C and S4A,B). Neuropathic pain mechanisms reportedly involve microglia only in male mice^5^, and we considered that the presence of a few female mice in the tested population might obscure some effect on mechanical hypersensitivity limited only to the males. However, when data from female animals were excluded in a separate analysis (*not shown*), we did not uncover any hidden male-specific effect of Panx1 deletion in either of these conditional knockout lines (n.s. for genotype or interaction by 2-way RM-ANOVA comparing ipsilateral values between *Panx1*^*fl/fl*^ and Cre(+) male littermates from CX3CR1-Cre or LysM-Cre lines, n = 6–9 and n = 8–9, respectively). This suggests that myeloid/macrophage loss of Panx1 alone is not sufficient to provide attenuation of SNI-induced mechanical allodynia.

We then tested whether the expression of Panx1 in the T lymphocyte lineage is necessary as T cells have also been linked to neuropathic pain^24^; this was achieved by crossing *Panx1*^*fl/fl*^mice to the CD4-Cre strain. However, CD4-Cre/*Panx1*^*fl/fl*^ mice developed the typical mechanical hypersensitivity despite elimination of Panx1 from lymphocytes (Figs 3E,F and S4C). To test this in another way, we performed adoptive transfer experiments, introducing T cells from *Panx1*^+/+^ or *Panx1*^−/−^ mice into T/B cell-deficient Rag1KO mice (Fig. S5A). Transferring Panx1-deficient T cells into Rag1KO mice failed to alter mechanical hypersensitivity following SNI (Fig. S5C,D). Of note, effective reconstitution of Rag1KO mice was achieved using T cells of either genotype (Fig. S5B).

We next asked whether Panx1 deficiency alters the inflammatory cell profile within neural tissues. Prominent within this profile are macrophages, which contribute to hypersensitivity early after injury in the periphery and then at later time points from within the CNS^3^,^4^,^24^. Resident macrophages (i.e., microglia in the CNS) as well as recruited circulating monocytes are believed to promote neuropathic pain. Indeed, at 7 days after injury, we observed a significant increase in the number of activated microglia in the ipsilateral spinal cord dorsal horn, relative to contralateral, as assessed by Iba1 immunostaining and characteristic morphological features (data not shown) or by co-immunostaining for Iba1 and phosphorylated p38 MAPK (pp38, an established marker of microglial activation^27^ (Fig. S6A,B). However, this ipsilateral increase in activated spinal microglia was fully retained in *Panx1*^−/−^ mice. In the periphery, a significant ipsilateral:contralateral increase in the number of macrophages was observed in the transected nerve (within 500 μm of the injured end) after injury in *Panx1*^+/+^ mice (Fig. S6C–E). Notably, whereas *Panx1*^−/−^ mice also had macrophage infiltration into the cut nerve, there was no significant difference between the ipsilateral and contralateral sides (Fig. S6C–E). Insofar as *Panx1*^−/−^ mice did not show the typical increase in macrophage infiltration, these data suggest that *Panx1*^−/−^ mice may have blunted neural inflammation after injury.

### A receptor-mediated Panx1 activation mechanism for SNI-induced hypersensitivity

Taking advantage of the rescue of a pain-like response obtained with bone marrow transplantation, we addressed whether different mechanisms of Panx1 activation can be linked to neuropathic pain after nerve injury. We focused on channel activation by caspase-mediated cleavage of the Panx1 C-terminal tail and by an intracellular loop mechanism required for G protein-coupled receptor activation, since caspases and various G protein-coupled receptors can contribute to neuropathic pain^3^,^24^,^28^, and because we have previously demonstrated that activation of Panx1 by either mechanism can be disrupted by specific Panx1 mutations^10^,^29^,^30^. For this, we re-introduced wild-type or mutant channels into *Panx1*^−/−^ hematopoietic cells by retroviral transduction to determine whether these modes of Panx1 activation affect SNI-induced hypersensitivity.

**Figure 4:**
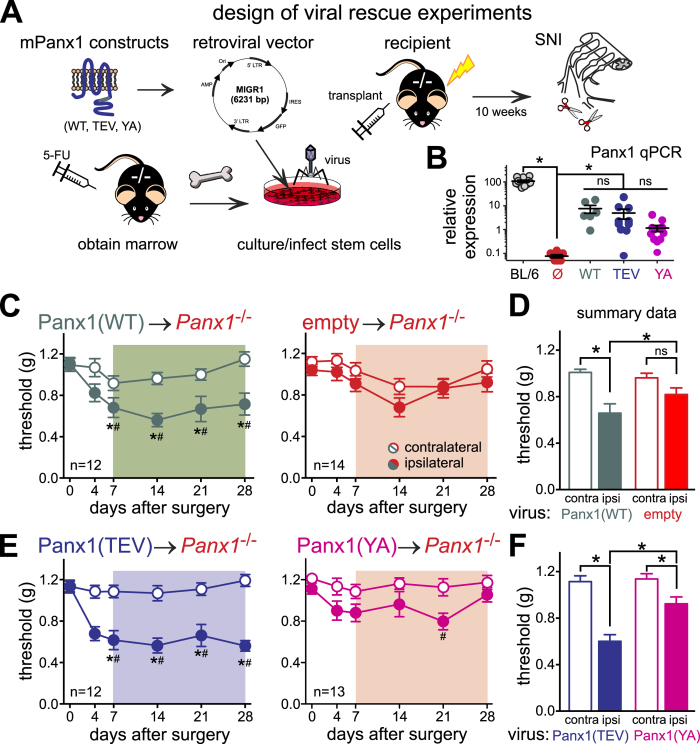
Restoration of Panx1 expression in adult *Panx1*^−/−^ hematopoietic cells restores pain following nerve injury; rescue requires tyrosine 198 of Panx1. (**A**) Study design^55^. (**B**) RT-qPCR for Panx1 (relative to HPRT) from bone marrow of uninfected C57BL/6 mice, or mice who received virally-infected hematopoietic cells; *p < 0.05 vs. empty vector. (**C,D**) Mechanical hypersensitivity develops in mice receiving cells infected with Panx1(WT), but fails to develop in empty-vector treated cells. (**E,F**) Mechanical hypersensitivity develops fully in mice receiving cells infected with Panx1(TEV), but is reduced in Panx1(YA) treated cells. Statistical analysis of behavioral data as in Fig. 1.

We obtained retroviral particles using an IRES-GFP vector (Migr1^31^) that were engineered to express: the GFP reporter alone (empty vector); wild-type mouse Panx1 (mPanx1(WT)); a mutated mPanx1 resistant to activation by caspase cleavage (mPanx1(TEV)^10^,^29^); or a mutated mPanx1 resistant to activation by G protein-coupled receptors (mPanx1Y198A^30^), here called mPanx1(YA)). As expected, transfection of HEK293 cells with each of these vectors resulted in Panx1-like currents (characteristic basal *I*-V properties and inhibition by carbenoxolone, CBX; Fig. S7). We cultured bone marrow cells from donor *Panx1*^−/−^ mice^31^, infected the cells with one of the 4 viruses described above, and injected those virally transduced cells into lethally irradiated *Panx1*^−/−^ mice (see Fig. 4A). At least ten weeks after reconstitution, we tested these mice in the SNI model and made several interesting observations. First, reintroduction of wild-type mPanx1 into Panx1-deficient hematopoietic cells and reconstitution of Panx1-deficient mice with these cells restored mechanical hypersensitivity after nerve injury to the *Panx1*^−/−^ mice (Fig. 4C,D). Based on flow cytometry analysis of GFP-expressing bone marrow cells after reconstitution, the infection appeared to affect several subtypes of cells equally (monocytes, T cells, B cells; data not shown). This recapitulated results obtained from transplanting wild-type bone marrow into Panx1-deleted mice. Second, *Panx1*^−/−^ mice reconstituted with mPanx1(TEV)-infected bone marrow cells showed mechanical hypersensitivity after SNI that was comparable to that seen with mPanx1(WT)-infected cells (Fig. 4E,F). This suggests that caspase-mediated activation of Panx1 is not playing a role in the pain elicited by SNI. Third, re-expression of mPanx1(YA) was unable to fully rescue mechanical hypersensitivity in *Panx1*^−/−^ mice following nerve injury, with essentially no reduction in mechanical threshold at 28 days (Fig. 4E,F). This implies a specific requirement for the Panx1 intracellular loop Tyr-198 residue that is critical for GPCR-mediated Panx1 activation^30^. Relative expression of Panx1 was low in bone marrow cells from transplanted mice, by comparison to wild type mice (Fig. 4B), and despite an apparent trend for lower expression in the mPanx1(YA)-transplanted mice, there was no statistically significant difference in levels of expression with any of the mPanx1 viruses. This suggests that different expression levels are unlikely to explain the contrasting behavioral outcomes obtained in mice transplanted with mPanx1(TEV) and mPanx1(YA) mutants. These data are thus consistent with a GPCR-mediated Panx1 channel activation mechanism in hematopoietic cells for nerve injury-induced mechanical allodynia.

### Pharmacological targeting of Panx1 is effective in treating hypersensitivity following nerve injury

We next asked whether pharmacologically inhibiting Panx1 function would be a viable therapeutic approach to diminish nerve injury-induced hypersensitivity. We treated wild-type mice with two chemically distinct channel blockers: CBX (a classical Panx1 blocker) and trovafloxacin (Trovan), a fluoroquinolone antibiotic that inhibits Panx1 in a voltage-dependent manner^17^. Although CBX has multiple additional effects, including inhibition of Connexin 43 (Cx43) gap junction channels that have been implicated in pain^12^,^32^, the only other known target of Trovan is a microbial gyrase/topoisomerase^33^; importantly, Trovan has no effect on Cx43 channel activity^17^.

**Figure 5:**
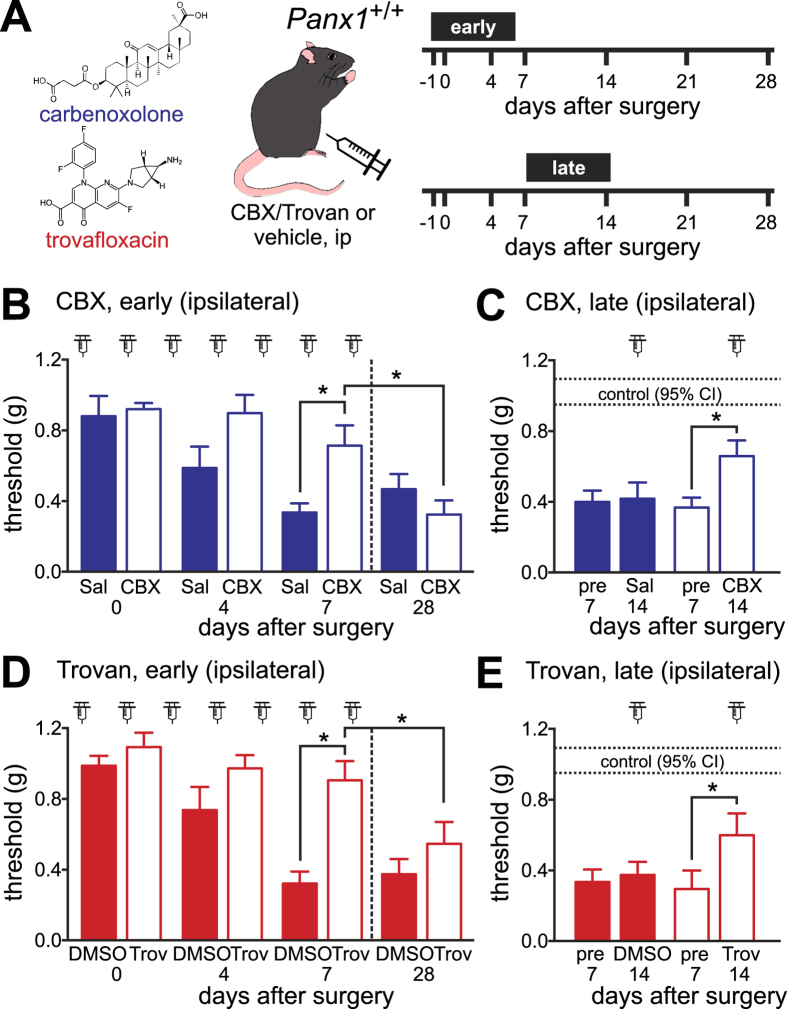
Pannexin channel inhibitors, carbenoxolone (CBX) and trovafloxacin (Trovan), prevent or reverse mechanical hypersensitivity when administered systemically. (**A**) Diagram depicting two dosing schemes. (**B,D**) The effect of early, daily IP CBX (**B**, 30 mg/kg in saline, n = 10–11 per condition) or Trovan (**D**, 30 mg/kg in DMSO, n = 10 per condition) and the respective vehicles. At day 7, pain development was significantly blunted by drug treatment, relative to vehicle; after the drug was withdrawn, full hypersensitivity was observed at day 28. (*p < 0.05 by two-way RM-ANOVA followed by Sidak’s multiple comparison test). (**C,E**) Changes in hypersensitivity after daily IP injections of CBX or saline (**C**, n = 12–13), and after Trovan or DMSO beginning at POD7 (**E**, n = 12–13). Mechanical sensitivity was partially relieved by both drugs, comparing pre- and post-treatment values (dotted lines: 95% CI for pre-operation baselines; *p < 0.05 by two-way RM-ANOVA followed by Sidak’s multiple comparison test).

In an early treatment protocol, CBX and Trovan were given systemically for 7 days beginning the day prior to SNI surgery (Fig. 5A). This early daily treatment with either CBX (Fig. 5B) or Trovan (Fig. 5D) resulted in complete protection from hypersensitivity; responses in ipsilateral paws of drug-treated mice were not different from pre-operation baselines for the period of drug administration. After discontinuation of the drug, however, hypersensitivity developed fully (Fig. 5B,D; see rightmost bars at day 28). We also tested if Panx1 blockers could relieve established allodynia in mice (late treatment, with drugs given daily starting 7 days after surgery; see Fig. 5A). Late treatment with both drugs partially relieved established hypersensitivity, recovering approximately half the decrease in mechanical threshold that followed nerve injury (Fig. 5C,E). It is unlikely that these effects were due to some unintended consequences of drug treatment (e.g., lethargy, or an inability to respond), since we found no drug-related effects on the performance of the mice using three different tests of motor function (Fig. S8). Thus, the observed effects of these drugs on mechanical threshold can be attributed to reduced hypersensitivity, suggesting that Panx1 inhibition may be a viable strategy for treating neuropathic pain.

## Discussion

In this study, we demonstrate that Panx1 is necessary for chronic mechanical allodynia following traumatic nerve injury, and Panx1 expression in hematopoietic cells is sufficient to account for its contribution in this model of neuropathic pain. Specifically, mice with global deletion of Panx1 fail to develop the typical mechanical and/or heat hypersensitivity that normally accompanies two different forms of sciatic nerve injury in mice, and hypersensitivity was rescued in Panx1-deleted mice transplanted with wild-type bone marrow cells. In addition, by transplanting *Panx1*^−/−^ bone marrow cells re-expressing wild-type or Panx1 constructs insensitive to specific forms of channel activation, we provide evidence that GPCR-mediated Panx1 activation in hematopoietic cells mediates these effects of the channel. Finally, systemic administration of two distinctly different Panx1 blockers prior to the injury was fully protective while later treatment partially relieved established hypersensitivity. Thus, Panx1 plays a major role in the development and maintenance of neuropathic pain following nerve injury and should be considered as a practicable therapeutic target for this often intractable condition.

Pharmacologic inhibition of Panx1 alleviated mechanical hypersensitivity when drugs were administered systemically. This suggests that targeting this channel may not require complicated spinal cord delivery methods^13^,^15^ and offers a more convenient potential translation toward clinical application. Since Trovan and CBX can both cross the blood-brain-barrier^34^,^35^ and hematopoietic cells gain access to the CNS after nerve injury^3^,^24^, systemic delivery of the blockers could modulate the channel either peripherally, at the site of nerve injury, or within the CNS. Our data also suggest that continued systemic administration of Panx1 blockers may be required for prolonged pain relief because hypersensitivity developed after the blocker was withdrawn. Encouragingly, however, attenuation of allodynia was achievable when blockers were provided after the establishment of that pain-like response, suggesting that treating patients already suffering from neuropathic pain with a Panx1 blocker may also prove effective.

Our bone marrow transplant studies highlight the importance of Panx1 in circulating hematopoietic cells for mechanical hypersensitivity following nerve injury. Together with complementary pharmacological studies in adult wild-type mice, this transplant work allays concerns with developmental compensation that can bedevil experiments using global knockout mice. Of particular note, even when transplanted bone marrow cells were initially derived from adult *Panx1*^−/−^ mice (i.e., cells that would have experienced any presumed developmental effects), it was possible to rescue the nerve injury-induced mechanical hypersensitivity in adult *Panx1*^−/−^ mice by acutely re-expressing wild-type Panx1 in the transplanted cells. Although the present results clearly attribute the major effect of Panx1 to its expression in circulating hematopoietic cells, we found that mechanical allodynia developed normally in injured mice depleted for Panx1 in various cell types commonly implicated in pain development^1^,^3^; aside from neurons and glia, this included hematopoietic cells of the macrophage/myeloid lineage and T cells. There are a number of possible explanations for these outcomes. For example, Panx1 may contribute via expression in a separate hematopoietic cell type that was not specifically targeted in our conditional knockout models (e.g., dendritic cells)^36^. Alternatively, there may be overlapping contributions among the targeted cell types (e.g., in amplifying or initiating innate inflammatory responses), or an emergent functional redundancy following Panx1 deletion from one cell type could allow a different cell type to compensate. Finally, it is possible that a low level of residual Panx1 expression after conditional deletion from one of these hematopoietic cell types is sufficient for development of hypersensitivity. Indeed, this possibility is supported by our viral re-expression experiments, where an average of ~7% of cells expressing Panx1 at ~5–10% of wild type levels was sufficient for restoration of the pain-like response. Thus, although Cre-mediated Panx1 deletion was clearly demonstrable in these conditional knockout models, Panx1 expression may have remained above a low threshold for effectiveness. Additional work will be required to understand how widespread expression of Panx1 in the various (and perhaps multiple) types of hematopoietic cells contributes to the complex interplay among these cells, and with elements of sensory pathways, that is envisaged in prevailing mechanistic models of neuropathic pain^1^,^3^.

Our data implicating Panx1 actions in hematopoietic cells urge reconsideration of earlier conclusions that Panx1 effects on pain were due to expression of the channel in spinal or sensory neurons^13^,^15^. This raises the question: Can the current data be reconciled with earlier results, if not the conclusions? First, it is important to point out that all Panx1 blockers used to date have non-specific effects, including the ^10^Panx peptide^12^. That issue notwithstanding, previous studies using intrathecal application do not preclude peripheral actions of the drugs; alternatively, hematopoietic cells can invade both the CNS and the dorsal root ganglia (DRG) after nerve injury^3^,^24^, so drug actions that occur in the CNS or DRG do not necessarily involve neurons directly. It was also reported that Panx1 is upregulated in DRGs after nerve injury, and that intrathecal delivery of Panx1 siRNA interfered with neuropathic pain^15^. Again, the DRG contains numerous non-neuronal cell types, including various inflammatory cell types that become more populous following nerve injury^3^,^24^, and it is therefore possible that measured injury-induced changes in Panx1 gene expression and/or effects of Panx1 siRNA on pain^15^ could be mediated by resident immune cells or infiltrating hematopoietic cells from the circulation. In short, the role for Panx1 that we observed in hematopoietic-derived cells is not necessarily inconsistent with those earlier data.

In mouse, Panx1 channels generate a modest constitutive current^29^, but further channel activation is required for Panx1 signaling, including ATP release^12^. Our experiments provide new insight into the mechanism of channel activation that is relevant for pain development. Specifically, we show that Panx1(Y198A) channels that are mutationally resistant to GPCR-mediated activation^30^, but not to caspase cleavage-based activation^10^,^29^, were not able to replicate the rescue of SNI-induced mechanical hypersensitivity observed with wild-type Panx1 following bone marrow transplantation in *Panx1*^−/−^ mice. Some caution is advised in interpreting these results since there was a trend, albeit non-significant, toward lower Panx1 expression in mice transplanted with Panx1(Y198A)-transduced cells; however, note also that low levels of Panx1 re-expression were obtained with all virally-transduced constructs even as only the Panx1(Y198A) construct failed to rescue mechanical allodynia.

A number of different Gq-linked receptors can activate Panx1^9^,^30^,^37^,^38^ and, where tested, the Y198A mutation abrogates receptor-mediated channel activation (e.g., by α1D adrenoceptors^30^ and histamine H1 receptors, unpublished data). Many of these Gq-linked receptors are also implicated in neuropathic pain, including the histamine H1 receptor^39^, purinergic receptors such as P2Y2^40^ and P2Y6^41^, and Group 1 metabotropic glutamate receptors^42^. P2Y6 and H1 receptors are of particular interest since they are prominently expressed in various immune cells^43^,^44^, and H1 receptor activation can induce Panx1-mediated ATP release^37^. Although it does not signal via a GPCR, TNFα has also been long-associated with neuropathic pain^45^ and it may activate Panx1 channels via the same Tyr-198 residue mutated in our experiments^8^. Similarly, it is also important to acknowledge that some alternative channel activating mechanism, not yet described, may also require an intact Tyr-198 residue on Panx1.

Other known channel activation mechanisms seem less likely. For example, Panx1 is described as a voltage-dependent channel, generating the largest currents at depolarized membrane potentials^6^,^12^. However, a depolarization-dependent activation mechanism is doubtful since Trovan is as effective as CBX at preventing or remediating neuropathic pain, even though Trovan blocks Panx1 channels only at hyperpolarized potentials^17^. As mentioned previously, activation of Panx1 channels by ionotropic receptors, specifically by P2X7 and NMDA receptors, has been proposed in the context of neuropathic pain^13^,^14^,^15^,^16^. However, it is not certain that Panx1 is actually the channel that underlies the P2X7-activated “large pore” conductance^46^. Moreover, a P2X7-mediated Panx1 channel mechanism is also unlikely since we found that our mouse lines express the C57BL/6 variant of P2X7 (P2X7Leu451, data not shown) that is unable to couple functionally to Panx1^16^. Although this precludes a requirement for P2X7 in the Panx1 activation relevant to nerve-injury induced pain, it does not rule out alternative mechanisms by which P2X7 could contribute to neuropathic pain. Finally, there is no direct evidence for the proposed NMDA-Panx1 mechanism in the context of neuropathic pain^14^ and although NMDA receptor activation can trigger Panx1 opening, this appears to be a Src-dependent process that targets a different tyrosine residue than the one mutated in our GPCR activation-resistant mutant^11^.

Collectively, these data provide compelling genetic and pharmacological evidence that Panx1 plays an obligatory role in the development and maintenance of chronic mechanical hypersensitivity following nerve injury that is linked to its expression in circulating bone marrow-derived cells. Panx1 channels are renowned for mediating release of ATP^12^, which is implicated in neuropathic pain^1^,^3^, but whether and how this (or another Panx1-permeant mediator) might contribute to Panx1-dependent chronic pain remains to be established. These studies also suggest that Panx1 is a potentially viable therapeutic target for prevention and/or alleviation of neuropathic pain, a condition that has been notoriously refractory to the current treatment options.

## Materials and Methods

### Reagents

Cell culture reagents were obtained from Life Technologies (Carlsbad, CA), subcloning reagents from New England Biolabs (Ipswich, MA), and all other reagents were purchased from Sigma Aldrich (St Louis, MO) unless specified otherwise below.

### Mice

Adult mice of both sexes were used in this study following procedures approved by the University of Virginia Animal Care and Use Committee (Protocol #2454), in adherence to the NIH Guide for the Care and Use of Laboratory Animals. All mice used in these experiments were on a C57BL/6 background. *Panx1*^−/−^ mice and *Panx1*^*fl/fl*^ mice were generated as previously described (Fig. S1A–C^17^). We obtained *Panx1*^+/+^ and *Panx1*^−/−^ littermates from heterozygous crosses; *Panx1*^+/−^ heterozygous littermates were not examined. The absence of Panx1 protein in the *Panx1*^−/−^ mice was verified by Western blot in various tissues (brain, Fig. S1C; thymus, liver, spleen, ear, heart, data not shown). To generate various conditional knockout lines, the following Cre-driver mice were used: GFAP-cre (Jackson Laboratories, stock#012886), Synapsin-Cre (Jackson Laboratories, stock#003966), LysM-cre (Jackson Laboratories, stock#004781), CX3CR1-cre (Jackson Laboratories stock#0025524^25^), CD4-cre (Taconic stock#4196). Conditional knockout lines were monitored frequently for germline deletion and, if observed, breeders were replaced. Because we observed that maintaining the Cre(+) allele exclusively on the female breeder greatly reduced the occurrence of germline deletion our breeding strategy for conditional knockouts involved crosses between *Panx1*^f/f^:Cre^+^ females and *Panx1*^f/f^ males. In some of the bone marrow transplant experiments, mice expressing GFP under the ubiquitin promoter were used to confirm transfer efficiency (UBI-GFP mice; Jackson Laboratories, stock#004353).

### Surgeries

To induce neuropathic pain, mice underwent spared nerve injury (SNI)^18^ or chronic constrictive injury of the sciatic nerve (CCI)^19^. Mice were anesthetized with isoflurane and received bupivacaine at the site of incision prior to surgery. After skin incision over the dorsal aspect of the upper thigh, a blunt dissection through the muscle allowed access to the sciatic nerve either at its branch point (for SNI) or proximal to the branch (for CCI). For SNI, the common fibular and tibial nerves were tightly ligated together using 6–0 sutures then cut to remove approximately 1 mm of nerve distal to the ligation point. The sural nerve was left completely intact and untouched. For CCI, the sciatic nerve was loosely ligated (until the first contraction of the distal muscles was observed) by two 4–0 silk sutures at approximately 5 mm from the emergence of the nerve from the greater sciatic foramen. Sham operations involved exposure of the nerve with no further manipulations. After nerve injury or sham procedure, the incision was closed by sutures and skin glue.

### Mechanical sensitivity

Sensitivity to touch was assessed using the standard up-down method, modified for mice, using von Frey filaments (Stoelting, Wood Dale, IL) of sizes 1.65 (0.008 gr of force) to 4.17 (1.4 gr of force)^47^. Mice were placed in clear plexiglass containers on an elevated wire mesh surface and allowed to acclimate at least 30 min prior to testing. Starting with the middle filament, the filaments were applied to the lateral portion of the hindpaw (for SNI) or central hindpaw (for CCI) until the filament just bent. A response was indicated by a sharp withdrawal of the paw. An average of three trials was obtained in order to calculate the final 50% response threshold per paw, per testing day. A minimum of 10 min of rest was allowed between each trial. Day “0” testing was an average of two baselines taken on separate days.

### Heat sensitivity

Sensitivity to heat was quantified by the Hargreaves method as the paw withdrawal latency to radiant heat (PWL, measured in seconds) as described^48^,^49^. The measurement system consisted of a clear plastic chamber (8 × 8 × 18 cm) sitting on an elevated clear glass floor that was temperature-regulated at 30 °C. Each mouse was placed in the chamber for 30 min to accommodate. A radiant heat source mounted on a movable holder beneath the glass floor was positioned to deliver heat focally to the plantar surface of either the right or left hindpaw. When the mouse withdrew the paw, the timer was shut off. To prevent thermal injury, the light beam automatically discontinued after 20 sec if the mouse failed to withdraw its paw. An average of four trials was obtained in order to calculate the PWL per paw on each day. A minimum of 10 min of rest was allowed between each trial. Day “0” testing was an average of two baselines taken on separate days.

### Sensorimotor testing

Mice administered drugs were monitored for motor proficiency and alertness using sensorimotor testing modified from a previously described method^48^,^49^. Three home-made apparatuses were used: an elevated ledge, an elevated platform and an inverted wire-mesh screen. The tests had a maximum cut-off time of 60 sec and were performed in the same order (ledge, platform, inverted screen). The elevated ledge was 22 cm high and 5 mm wide. The maximum score was given if the mouse traversed the entire length of the ledge (32 cm) within the allotted time or maintained its balance on the ledge for that same period of time. The platform test used an elevated circular platform of 35 mm diameter and at a height of 31 cm. The length of time a mouse remained on the platform was measured. The maximum score was given if the mouse remained on the platform for 60 sec or if it climbed down the pole holding the platform. For the inverted screen test, the mouse was placed on a wire mesh grid measuring 17 × 50 cm (15 squares per 10 cm) and the grid was rapidly inverted so the mouse was hanging upside-down. The mouse was timed to see how long it could hang on the screen. The area underneath each apparatus was heavily padded to prevent injuries from potential falls and the mouse was allowed at least 5 min of rest between each apparatus.

### Adoptive Transfer

Donor mice were sacrificed by CO_2_ exposure, and lymph nodes and spleens were rapidly dissected into Phosphate-buffered saline (PBS) with 10% fetal bovine serum (FBS). Single cell suspensions were made by pressing the tissue through 70 μm nylon mesh and T cells separated by negative selection using a Pan T cell Isolation Kit (Miltenyl Biotech, San Diego, CA). Cells were resuspended in Roswell Park Memorial Institute (RPMI) media and 2 × 10^6^ cells were given via tail vein injection to each adult Rag1KO mouse (Jackson Laboratories stock#00216). Mice underwent SNI 10 days after the transfer. After injury, spleens and lymph nodes were collected from some of the mice to confirm the efficiency of the reconstitution by Fluorescence-activated cell sorting (FACS).

### Bone marrow transplants

Donor mice were sacrificed by CO_2_ exposure, hindlimb bones extracted and flushed with PBS. The bone marrow cells were washed, filtered through 70 μm mesh and resuspended in PBS so that each recipient mouse received 3 × 10^6^ cells via tail vein injection. Recipient mice were irradiated with 2 doses of 600 rads using a Shepherd Mark irradiator. The irradiations occurred on the same day separated by 4 h and injections of new bone marrow cells were given approximately 24 h after the final irradiation. The recipients were maintained on antibiotic water (80 mg/mL sulfmethoxazole and 0.37 mM trimethoprim) for one week before and four weeks after irradiation. All experiments, including SNI surgeries, occurred at least ten weeks after the irradiation.

### Generation of viral constructs and particles

Viral vectors for expression of Panx1 in bone marrow cells were sub-cloned into Migr1, a vector previously described for *ex vivo* infection of bone marrow cells (ref. 31; Addgene plasmid #27490). Briefly, pcDNA3.1 vectors containing the three versions of Panx1 (Panx1(WT), Panx1(TEV), Panx1(YA)) were cut with HindIII, incubated with DNA polymerase I (Klenow) to produce blunt ends then digested with EcoRI. Migr1 (cut with XhoI then incubated with DNA polymerase I followed by digestion with EcoRI) was ligated with the appropriate gel-extracted band from the pcDNA3.1 digest. After transformation of One Shot Stbl3 competent cells (ThermoFisher, Waltham, MA), plasmids were purified and sequenced. The final vectors were packaged by the University of Michigan Vector Core using transient co-transfection of the plasmids pUMVC-Gag/Pol (Aldevron, Fargo, ND, and the University of Michigan vector core) and pCl-VSVg envelope (Addgene #1733).

### Virally infected bone marrow chimeras

Methods for *ex vivo* infection of bone marrow cells and reconstitution in irradiated mice were modified from a previously described method^31^. Donor *Panx1*^−/−^ mice were pretreated with 2 mg of 5-fluorouracil for 4 days prior to bone marrow harvest. The red blood cells were lysed, and the remaining cells resuspended at a concentration of 1 × 10^6^ cells per ml and cultured overnight in a 37 °C humidified incubator with 5% CO_2_. Media consisted of Isocove’s Modified Dulbecco’s Media supplemented with 15% FBS, 2 mM glutamax, 1% penicillin/streptomycin, 50 μM β-mercaptoethanol, 5 ng/mL IL3 (Peprotech, Rockyhill, NJ), 5 ng/mL IL6 (Peprotech) and 50 ng/mL of stem cell factor (R&D Systems, Minneapolis, MN). Cells were replated the next day with the inclusion of 4 mg/mL polybrene (Millipore, Darmstadt, Germany) and at a density of 2 × 10^6 ^cells per mL. Spin innoculation then occurred at 1300G and 20 °C in a table-top centrifuge for 2 h using 1 mL of virus concentrate per 3 mL of cells. Cells were then returned to the incubator overnight and the spin inoculation was repeated the next day. After 16 h, the cells were resuspended in PBS and injected via tail vein to lethally irradiated mice (see above for details) at a minimum of 500,000 cells per mouse.

### Isolation and enrichment of peritoneal macrophages

Adult *LysM-Cre::Panx1*^*fl/fl*^ and *CX3CR1-Cre::Panx1*^*fl/fl*^ mice of either genotype (i.e., Cre-positive or Cre-negative) were euthanized by CO_2_ exposure and injected with 10 mL PBS delivered via a 28G needle into the peritoneal cavity immediately after confirmation of death. The fluid was collected via a 23G needle and the cells washed, resuspended in Dulbecco’s Modified Eagle Media: Nutrient Mixture F12 (DMEM/F-12 media, supplemented with 10% FBS, 10 mM L-glutamine, 1% penicillin/streptomycin), plated and incubated at 37 °C with 5% CO_2_ for 3 days. The media was changed daily and on the last day, cells were lysed in the 6-well plate and the exudate collected for RT-qPCR analysis.

### Isolation and enrichment of astrocytes

4 to 6 day-old pups of the *GFAP-Cre::Panx1*^*fl/fl*^ line were sacrificed by rapid decapitation under ketamine-xylazine anesthesia (375 and 25 mg/kg i.m.). Cortices were dissected into Hank’s Balanced Salt Solution, meninges removed and the tissue minced into 1 mm pieces. Brains were then digested in 0.25% trypsin at 35 °C for 30 min followed by gentle trituration of the tissue in glass pipettes of increasingly smaller sizes. The resulting suspension was filtered through a 70 μm mesh and astrocytes were enriched using an anti-GLAST microbead kit (Miltenyl Biotech) and the positive-selection procedure on an AutoMacs Pro machine (Miltenyl Biotech) according to the manufacturer’s protocol. This procedure yielded approximately 2-fold enrichment for astrocytes in the final positive selection as assessed by RT-qPCR for genes specific to astrocytes and other brain cells (neurons and microglia, details listed below; data not shown).

### Isolation and enrichment of neurons

5 day-old pups of the *Syn-Cre::Panx1*^*fl/fl*^ line were sacrificed by rapid decapitation under ketamine-xylazine anesthesia (375 and 25 mg/kg i.m.). Whole brains were dissected into DMEM/F12 media, meninges removed and the tissue minced into 1 mm pieces. Brains were then digested in 120 U/mL papain (Worthington Biochemical Corporation) at 30 °C for 30 min followed by gentle trituration of the tissue in glass pipettes of increasingly smaller sizes. The resulting cells were spun at 200G for 4 min, resuspended in Neurobasal media supplemented with 2% B27 and 0.5 mM Glutamax. Cells were plated at a concentration of 1 × 10^6^ cells per mL on poly-l-lysine coated 6-well plates with partial media changes every 4 days. After 8 days, the cells were incubated for 48–72 h with 1 μM cytosine β-D-arabinofuranoside hydrochloride to inhibit the growth of any contaminating cells. Cells were lysed directly in the 6-well plate and the exudate collected for RT-qPCR analysis. This procedure generates semi-purified neuronal cultures (~50% neuronal cells^50^,^51^).

### Isolation and enrichment of T cells

Adult *CD4-Cre::Panx1*^*fl/fl*^ mice were sacrificed by CO_2_ exposure, and lymph nodes and spleens were rapidly dissected into PBS with 10% FBS. Single cell suspensions were made by pressing the tissue through 70 μm nylon mesh and T cells separated by negative selection using a Pan T cell Isolation Kit (Miltenyl Biotech). Cells were pelleted, lysed and saved for further RT-qPCR analysis.

### Quantitative real-time PCR

Cells were isolated by various methods as described above. mRNA was extracted using a Trizol reagent and a RNeasy Mini Kit (Qiagen, Hilden, Germany). Equal quantities of mRNA were reverse transcribed using the iScript cDNA synthesis kit (Biorad, Hercules, CA). 25–100 ng of cDNA was then added to a 96-well plate in triplicates and analyzed using a CFX Connect Real-Time PCR Detection System (Biorad). For expression of Panx1, Taqman probes and iTaq Universal Probes Supermix was used (ThermoFisher, probe Mm00450900_m1 for Panx1, probe Mm01545399_m1 for HPRT, and probe Mm99999915_g1 for GAPDH). To assess the efficiency of various cell isolation methods, iQ SYBR green Supermix (Biorad) and primers for the following transcripts were used (Supplementary file 1): Slc1a3 for astrocytes, Itgam for microglia, and Map2 for neurons and β-actin or GAPDH as housekeeping genes. Template and primer dilutions, melt curves and amplicon sizes were used to verify primer specificity. For all RT-qPCR data, cycles to threshold were analyzed using a 2^−ΔCt^ normalization procedure^52^.

### Immunohistochemistry

Mice were anesthetized, perfused with PBS followed by 4% paraformaldehyde (PFA) and tissue removed. For analysis of spinal cord sections, the tissue was left in PFA for two additional nights then 30 μm sections were cut from the lumbar prominence on a vibrating microtome (VT1000s, Leica Microsystems, Wetzlar Germany). Free-floating sections were washed in Tris-buffered saline (TBS), blocked in 10% normal horse serum then incubated with rabbit anti-pp38 (Cell Signaling Technologies, Danvers, MA; 1:1000) for two nights at 4 °C followed by incubation with unconguated goat anti-rabbit (Jackson ImmunoResearch, West Grove, PA; 1:50 for 90 min at room temperature) and finally FITC conjugated anti-goat secondary antibody (Jackson ImmunoResearch; 2 h at room temperature). A second primary antibody of rabbit anti-Iba1 (Wako, Osaka, Japan; 1:600) was then used for two nights at 4 °C followed by rhodamine conjugated anti-rabbit secondary antibody (Jackson ImmunoResearch; 2 h at room temperature). Sections were then dehydrated, mounted on glass slides and cover-slipped.

For peripheral nerve analysis, tissues were post-fixed overnight and embedded in paraffin. 5 μm thick longitudinal sections were mounted on slides, which were deparaffinized and underwent either haematoxylin and eosin (H&E) staining or immunohistochemistry. For immunohistochemistry, antigen retrieval occurred via 10 mM sodium citrate (pH 6.0) in a pressure cooker for 30 min. Slides were then washed, blocked in 10% normal horse serum in TBS (2 h at room temperature), endogenous peroxidases blocked with 0.3% H_2_O_2_ and incubated overnight in rabbit anti-Iba1 (1:500). The next day, the slides were incubated with secondary antibody (biotinylated anti-rabbit, 1:1000 for 2 h, Jackson ImmunoResearch) followed by processing with Elite ABC reagents (Vector Laboratories, Burlingame, CA) and a DAB substrate kit (Vector Laboratories). Slides were finally dehydrated and cover-slipped.

### Toluidine blue analysis

Mice were anesthetized, perfused with PBS followed by 4% PFA and the distal segments of the sciatic nerve extracted. After overnight fixation at 4 °C, samples were then postfixed in 1% osmium tetroxide, dehydrated in a series of graded alcohols and embedded in epon. Semithin (0.5 μm) transverse sections were cut on an ultramictrome (Leica Ultracut), stained with toluidine blue and cover-slipped.

### Image acquisition and analysis

Investigators blinded to genotype and/or experimental condition examined slides under an epifluroescence microscope (Zeiss Axioimager Z1; Oberkochen, Germany) with motorized stage using Neurolucida software (MBF Bioscience, Williston, VT). For Iba1-facilitated analysis of microglia activation, the morphology of the cells allowed for determination of activation state, where activated cells had rounded cell bodies and short processes^53^. For analysis of the injured sciatic nerve, the longitudinal slice was considered representative of the injury if the H&E stained section adjacent to it demonstrated signs of injury (red blood cells, tissue fibrous, and Iba1-postive macrophages surrounding the cut end); only tissue enclosed by the perineural sheath and within 500 μm of the end was analyzed. For toludine blue analysis, nerves were counted as intact profiles if the myelin appeared as a continuous, round structure with a clear axon center; nerves were counted as degenerating profiles if the cross-section was a myelin ovoid or it appeared as a dark, irregular profile that lacked axoplasm. Data are presented as average values of 3–5 slices for dorsal horn sections and 1–2 representative section for peripheral nerves.

### Flow Cytometry

Mice were sacrificed by CO_2_ exposure and the following tissues collected for flow cytometry analysis: blood was collected via cardiac puncture, followed by immediate lysis of red blood cells; spleens were rapidly dissected into RPMI with 10% FBS, and the bone marrow was collected by flush using a 23G needle (BD Biosciences, Franklin Lakes, NJ). Single cell suspensions were made by pressing the tissues through a 70 μm nylon mesh (ThermoFisher). Cellular composition and expression of GFP was analyzed after staining with antibodies specific for CD3, CD4, CD8, CD19, Thy1.2, CD11b, Ly6C and Ly6G. All antibodies used were obtained from eBioscience (San Diego, CA). Flow cytometry was performed using FACSCanto II (BD Biosciences) and the results were analyzed with the FlowJo software (TreeStar Inc., Ashland, OR).

### Electrophysiology

HEK293T cells were transfected with mPanx1 retroviral targeting plasmids (Panx1(WT), Panx1(TEV) and Panx1(YA)) using Lipofectamine 3000 (Invitrogen, Carlsbad, CA). After ~12h, cells were transferred on coverslips, allowed to attach and used for whole cell patch clamp recordings. To isolate Panx1 currents, the extracellular solution contained (in mM): 140 NaCl, 3 KCl, 2 MgCl_2_, 2 CaCl_2_, 10 glucose and 10 HEPES (pH 7.3). The internal (pipette) solution contained (in mM): 100 CsMeSO_3_, 30 tetraethylammonium chloride (TEA-Cl), 4 NaCl, 1 MgCl_2_, 0.5 CaCl_2_, 10 EGTA, 3 ATP-Mg, 0.3 GTP-Tris and 10 HEPES (pH adjusted to 7.3 with CsOH). Whole cell currents were measured using an Axopatch 200B, Digidata 1440A and pClamp acquisition software (all electrophysiology equipment purchased from Molecular Devices, Sunnyvale, CA). Whole cell currents were recorded while applying a voltage ramp protocol (−100 mV to 80 mV in 1.5s; holding potential −50 mV). Extracellular solution containing CBX (50 μM) was perfused after stabilization of the outwardly-rectifying Panx1 currents. The acquired signals were filtered at 1 kHz and sampled at 2 kHz for digitization. Data were normalized to cell capacitance (from the amplifier circuits) and quantified as current density (pA/pF) at + 80 mV and −60 mV. All recordings were carried out at room temperature.

### Western blots

Cerebral cortices of mice were dissected in cold dissection buffer (26 mM NaHCO_3_, 3 mM KCl, 1.25 mM NaH_2_PO_4_, 10 mM glucose, and 2 mM MgCl_2_) and lysed in a protein extraction buffer (50 mM Tris, 150 mM NaCl, pH 7.4, 1% Triton X-100, 0.2% SDS, a cocktail of protease inhibitors, 5 mM NaF, and 10 mM NaVO_3_) by using TissueLyser II (Qiagen). Protein samples were separated by SDS-PAGE, transferred onto 0.45 μm nitrocellulose membranes (Amersham) and blocked with 5% non-fat dry milk dissolved in a tris-based buffer (10 mM Tris, 150 mM NaCl, and 0.1% Tween 20, pH 7.4) at room temperature for 1 h. Endogenously expressed Panx1 proteins were detected by incubating with anti-Panx1 antibodies (C-terminus^54^), and anti-α-tubulin (T9026, Sigma Aldrich) antibodies were used as loading controls. Amersham ECL horseradish peroxidase (HRP)-linked secondary antibodies (GE Healthcare, Little Chalfont, United Kingdom) and Amersham ECL Western blotting detection reagent were used to visualize immunoreactive signals on Amersham Hyperfilm ECL (GE Healthcare).

### Statistics

All behavioral data and image analysis was conducted by experimenters blinded to animal genotype or experimental condition. Data are presented as means ± s.e.m, with p < 0.05 considered statistically significant for the indicated tests. Raw, untransformed, time-course data are presented for all post-injury behavioral analyses, with statistical analyses by two-way RM ANOVAs. Summary data were obtained by averaging data from each mouse between POD7-28, and treating that average as a single point in determining group means for analysis by two-way RM ANOVA.

## Additional Information

**How to cite this article**: Weaver, J. L. *et al*. Hematopoietic pannexin 1 function is critical for neuropathic pain. *Sci. Rep.* **7**, 42550; doi: 10.1038/srep42550 (2017).

**Publisher's note:** Springer Nature remains neutral with regard to jurisdictional claims in published maps and institutional affiliations.

## Supplementary Material

Supplementary Data
