# Supplementary material for: Hematopoietic pannexin 1 function is critical for neuropathic pain
Source: Sci Rep. 2017 Feb 14;7:42550. doi: 10.1038/srep42550 (PMC5307344; doi:10.1038/srep42550)
Supplement: Supplementary Data [file srep42550-s1.pdf]

Supplementary Material

Hematopoietic pannexin 1 function is critical for neuropathic pain

Janelle L. Weaver[1], Sanja Arandjelovic[2,3,4], Gregory Brown[1], Suresh Mendu[1], Michael Schappe[1], Monica W. Buckley[2,3,4], Yu-Hsin Chiu[1], Shaofang Shu[1], Jin K. Kim[1], Joyce Chung[1], Julia Krupa[1], Vesna Jevtovic-Todorovic[5], Bimal N.Desai[1], Kodi S. Ravichandran[2,3,4], and Douglas A. Bayliss[1]*

[1] Department of Pharmacology, University of Virginia, Charlottesville, Virginia 22908, USA.

[2] Department of Microbiology, Immunology, Cancer Biology, University of Virginia, Charlottesville, Virginia 22908, USA.

[3] Carter Immunology Center, University of Virginia, Charlottesville, Virginia 22908, USA.

[4] Center for Cell Clearance, University of Virginia, Charlottesville, Virginia 22908, USA.

[5] Department of Anesthesiology, University of Colorado, Aurora, Colorado 80045 USA.

*Correspondence to: Douglas A. Bayliss, Department of Pharmacology, University of Virginia Health System, 5009A Jordan Hall, PO Box 800735, 1340 Jefferson Park Avenue, Charlottesville, Virginia, USA 22908-0735; phone: (434) 982-4449, e-mail: bayliss@virginia.edu

Supplementary Material: Figures S1-S8

Table S1

Supplementary Figure Legends

Supplementary Figure 1. Development and properties of Panx1-deleted mice. (**A**) Targeting strategy for conditional and deleted Panx1 alleles. (**B**) PCR genotyping of wild-type, deleted and floxed alleles. (**C**) Western blot of brain tissue depicting loss of Panx1 protein in *Panx1*$^{-/-}$ mice. (**D**) Sensorimotor function of wild-type (n=14) and *Panx1*$^{-/-}$ mice (n=8) in tests on elevated ledge, elevated platform and inverted wire-mesh screen; there was no difference in performance between genotypes (n.s., p>0.36 for genotype in each assay by Mann-Whitney test; maximum time for each assay was 60s). (**E,F**) *Panx1*$^{-/-}$ mice have normal heat (**E**, n=10-21) and mechanical (**F**, n=25-43) sensitivity in the absence of injury (n.s., p>0.87 for genotype by two-way ANOVA).

Supplementary Figure 2. Panx1-dependent effects on SNI-evoked mechanical hypersensitivity are observed in both male and female mice and CCI-evoked heat hypersensitivity is blunted in *Panx1*$^{-/-}$ mice. (**A**) Mechanical sensitivity after SNI in male and female mice that were either *Panx1*$^{-/-}$ or *Panx1*$^{-/-}$ mice; data from Figure 1C, normalized to baseline (day 0). (**B**) Summary data showing effects of Panx1 deletion on mechanical threshold in male and female mice. (**C,D**) Heat sensitivity after CCI in wild-type (n=16) and *Panx1*$^{-/-}$ mice (n=14). (**E**) Example semithin sections of the sciatic nerve distal to the CCI suture placement 7 days after surgery. Scale bar in left image is 10 µm (**F**) Quantification of intact axonal density (above) and degenerating neuronal profiles (below; p>0.29 for both parameters by unpaired two-tailed t-test). Statistical analysis of behavioral data as in Figure 1.

Supplementary Figure 3. Expression of Panx1 in neurons or astrocytes alone is not required for neuropathic pain. (**A,B**) The development of mechanical hypersensitivity in *Panx1*$^{fl/fl}$ mice or GFAP-Cre littermates (n=12-13). (**C**) RT-qPCR for Panx1 relative to GAPDH from acutely isolated astrocytes (n=4-8, * p<0.05 by Kruskal-Wallis test followed by Dunn's multiple comparisons test). (**D,E**) The development of mechanical hypersensitivity in *Panx1*$^{fl/fl}$ mice or Syn-Cre littermates (n=9-14). (**F**) RT-qPCR for Panx1 relative to GAPDH from semi-purified neuronal cultures (n=3-8, * p<0.05 by unpaired two-tailed t-test). Statistical analysis of behavioral data as in Figure 1.

Supplementary Figure 4. Development of SNI-induced mechanical hypersensitivity in myeloid/microglia and T cell conditional knockout mice. (**A-C**) Time course for development of mechanical hypersensitivity in *Panx1*$^{fl/fl}$ mice or their cognate littermates from the CX3CR1-Cre line (**A**), the LysM-Cre line (**B**) or the CD4-Cre line (**C**). Statistical analysis as in Figure 1.

Supplementary Figure 5. Reintroduction of T cells from wild-type or *Panx1*$^{-/-}$ mice into T cell-deficient Rag1 knockout mice does not affect development of SNI-induced mechanical hypersensitivity. (**A**) Design of T cell adoptive transfer experiments. (SNI image adapted from [55]) (**B**) Representative cytometry plots of CD4 (APC) and CD8 (PE) staining of Thy1.2 positive splenocytes isolated from adoptive transfer mice after SNI. (**C,D**) SNI-induced mechanical hypersensitivity in Rag1KO mice after vehicle treatment (*left*) or after reconstitution with T cells from *Panx1*$^{+/+}$ (*middle*) or *Panx1*$^{-/-}$ mice (*right*). Statistical analysis of behavioral data as in Figure 1.

Supplementary Figure 6. *Panx1*$^{-/-}$ mice have lessened peripheral inflammation after nerve injury. (**A**) Example co-staining between Iba1 and pp38 in the dorsal horn of mice after 7 days of injury. Inset on left depicts a typical resting pp38(-) microglia, whereas inset on right shows a pp38(+), activated microglia. Scale bar is 25 µm. (**B**) Quantification of cells with an activated, amoeboid-like Iba1 staining with co-localization of pp38 (*, p<0.05 by two-way ANOVA followed by Sidak's multiple comparisons, n=5-6). (**C**) Example H&E staining of the injured sciatic nerve (left) and Iba1 staining (right). Note the effusion of red blood cells and other cells surrounding the nerve on the H&E stain, which was used to identify the cut end of the nerve. The red border depicts the region within the nerve used for further quantification. Scale bar on the larger images is 100 µm and on the higher magnification image is 25 µm. (**d**) Example high powered images taken from the boxed region in panel (**C**). Arrows are example Iba1(+) cells. (**E**) Quantification of macrophages within the nerve ending. (*, p<0.05 by two-way ANOVA followed by Sidak's multiple comparisons, n=5-8).

Supplementary Figure 7. Expression of Panx1 from retroviral vectors in HEK cells. (**A-C**, left) *I*-V curves of Panx1 currents from (**A**) mPanx1(WT), (**B**) mPanx1(TEV), and (**C**) mPanx1(YA) before and after 50 µM CBX application. (**A**-**C**, right) Summary data of current densities with and without CBX for each vector calculated at +80 mV and at -60 mV. Note that the mouse variant of Panx1 generates basal currents in the absence of activation [28].

Supplementary Figure 8. Mice treated daily with Panx1 blockers maintain sensorimotor capabilities. (**A-C**) Sensorimotor function of mice after 7 days of drug (T, trovan or C, CBX) or vehicle treatment (D, DMSO or S, saline). There were no differences in performance between SNI-operated mice receiving no treatment (null symbol) or any of the 7 day drug or vehicle treatments (p>0.06, Kruskal-Wallis test). In the last test (**C**, inverted screen), sham mice completed the task better than any of the SNI-operated mice (* p<0.05 by Kruskal-Wallis test followed by Dunn's multiple comparisons test).

Supplementary Figure 1

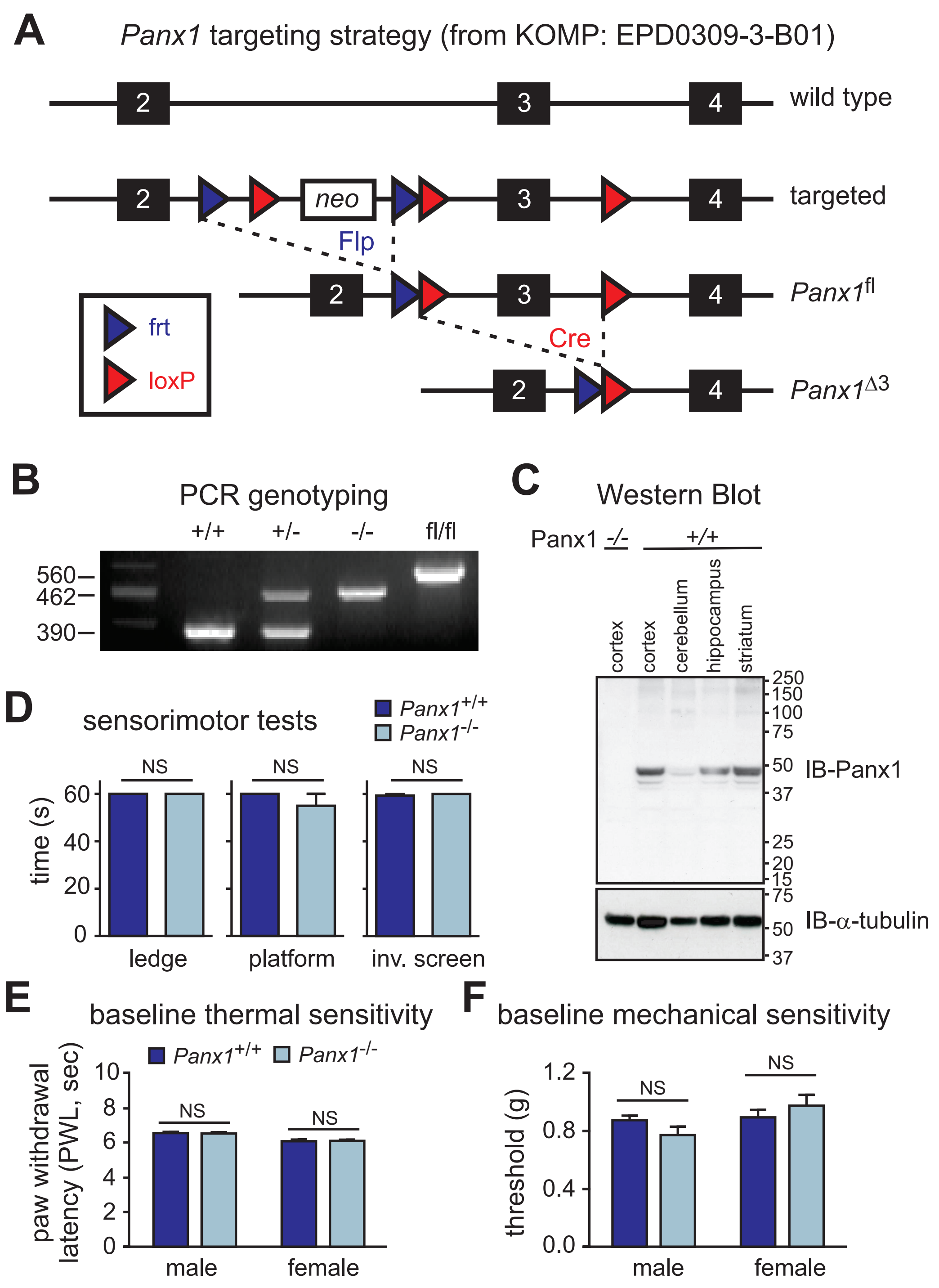

Figure S1. Development and properties of Panx1-deleted mice. (**A**) Targeting strategy for conditional and deleted Panx1 alleles. (**B**) PCR genotyping of wild-type, deleted and floxed alleles. (**C**) Western blot of brain tissue depicting loss of Panx1 protein in *Panx1*$^{-/-}$ mice. (**D**) Sensorimotor function of wild-type (n=14) and *Panx1*$^{-/-}$ mice (n=8) in tests on elevated ledge, elevated platform and inverted wire-mesh screen; there was no difference in performance between genotypes (n.s., p>0.36 for genotype in each assay by Mann-Whitney test; maximum time for each assay was 60s). (**E,F**) *Panx1*$^{-/-}$ mice have normal heat (**E**, n=10-21) and mechanical (**F**, n=25-43) sensitivity in the absence of injury (n.s., p>0.87 for genotype by two-way ANOVA).

Supplementary Figure 2

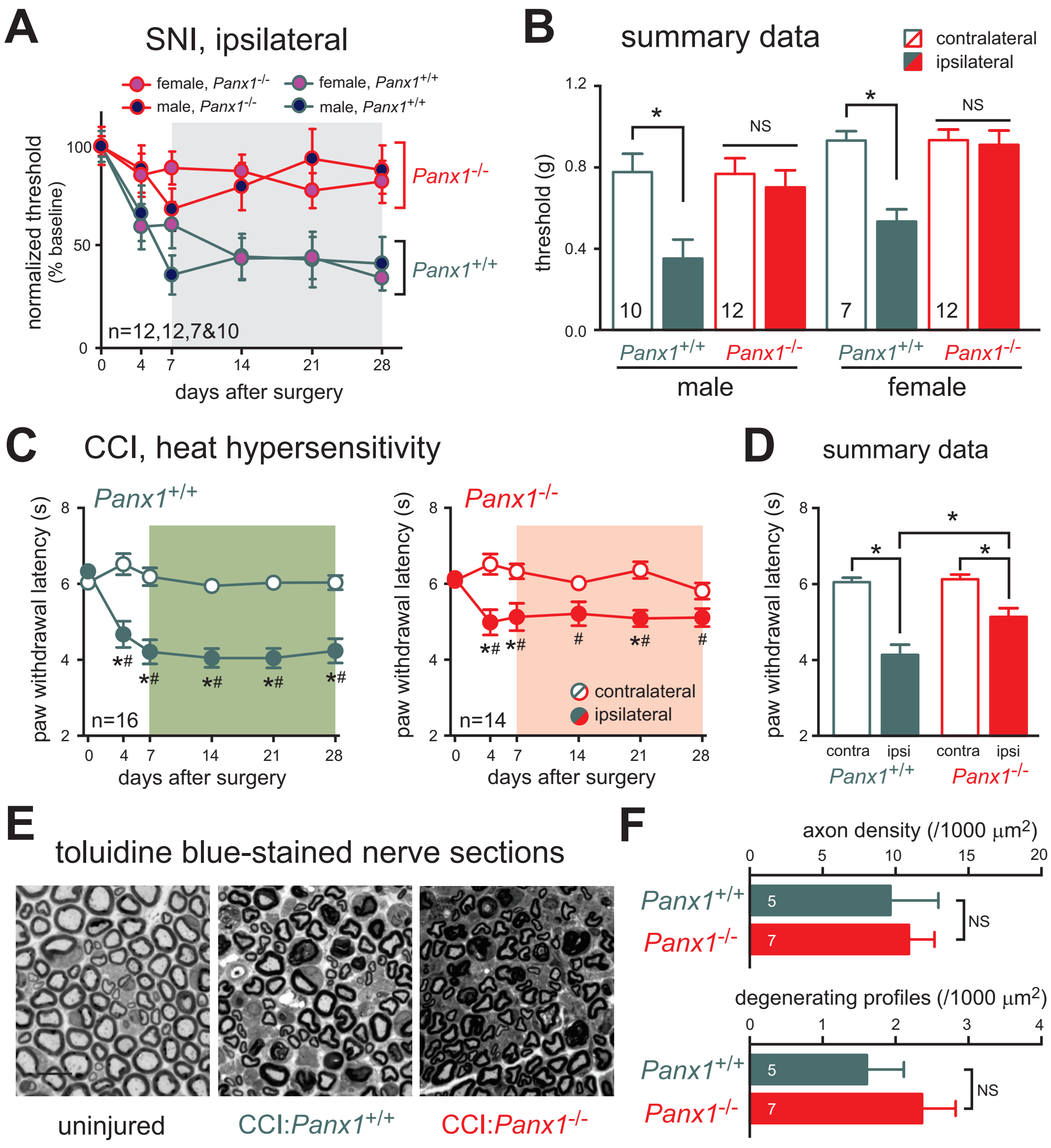

Figure S2. Panx1-dependent effects on SNI-evoked mechanical hypersensitivity are observed in both male and female mice and CCI-evoked heat hypersensitivity is blunted in *Panx1*$^{-/-}$ mice. (**A**) Mechanical sensitivity after SNI in male and female mice that were either *Panx1*$^{-/-}$ or *Panx1*$^{-/-}$ mice; data from Fig. 1c, normalized to baseline (day 0). (**B**) Summary data showing effects of Panx1 deletion on mechanical threshold in male and female mice. (**C,D**) Heat sensitivity after CCI in wild-type (n=16) and *Panx1*$^{-/-}$ mice (n=14). (**E**) Example semithin sections of the sciatic nerve distal to the CCI suture placement 7 days after surgery. Scale bar in left image is 10 μm (**F**) Quantification of intact axonal density (above) and degenerating neuronal profiles (below; p>0.29 for both parameters by unpaired two-tailed t-test). Statistical analysis of behavioral data as in Fig. 1.

Supplementary Figure 3

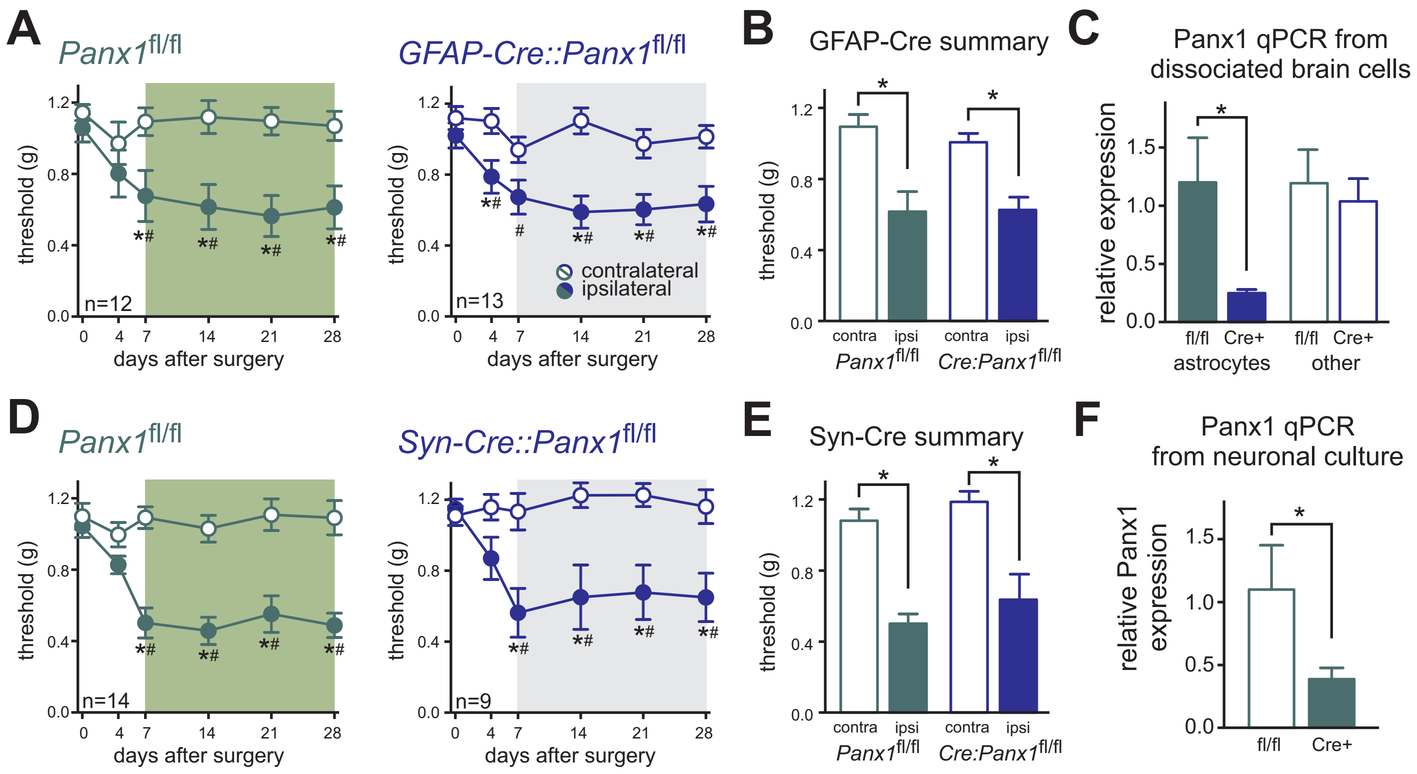

Figure S3. Expression of Panx1 in neurons or astrocytes alone is not required for neuropathic pain. (**A,B**) The development of mechanical hypersensitivity in *Panx1$^{fl/fl}$* mice or GFAP-Cre littermates (n=12-13). (**C**) RT-qPCR for Panx1 relative to GAPDH from acutely isolated astrocytes (n=4-8, * p<0.05 by Kruskal-Wallis test followed by Dunn's multiple comparisons test). (**D,E**) The development of mechanical hypersensitivity in *Panx1$^{fl/fl}$* mice or Syn-Cre littermates (n=9-14). (**F**) RT-qPCR for Panx1 relative to GAPDH from semi-purified neuronal cultures (n=3-8, * p<0.05 by unpaired two-tailed t-test). Statistical analysis of behavioral data as in Fig. 1.

Supplementary Figure 4

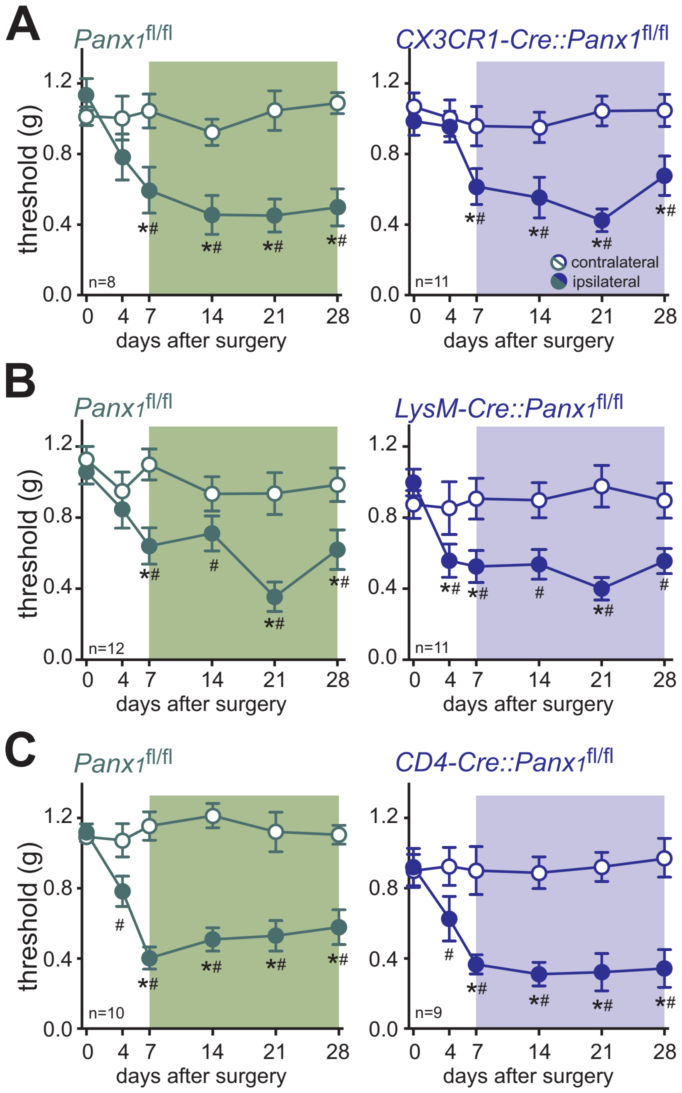

Figure S4. Development of SNI-induced mechanical hypersensitivity in myeloid/microglia and T cell conditional knockout mice. (**A-C**) Time course for development of mechanical hypersensitivity in *Panx1$^{fl/fl}$* mice or their cognate littermates from the CX3CR1-Cre line (**A**), the LysM-Cre line (**B**) or the CD4-Cre line (**C**). Statistical analysis as in Fig. 1.

Supplementary Figure 5

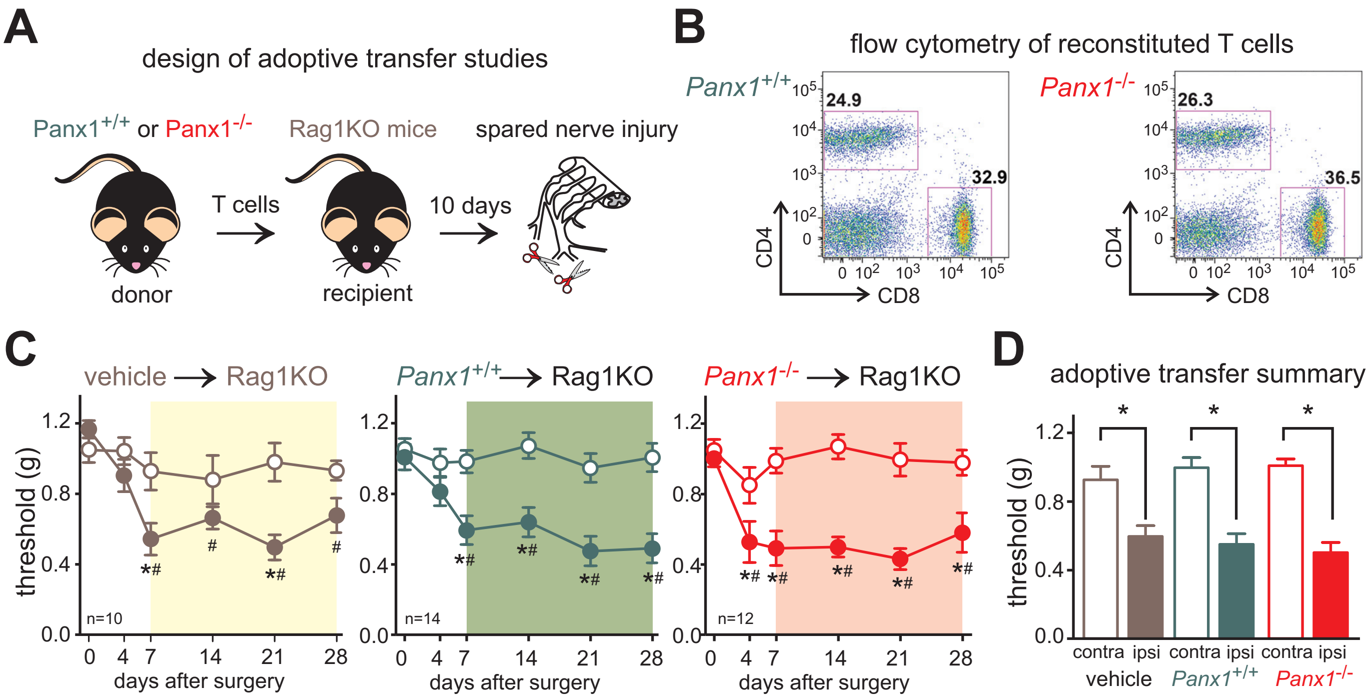

Figure S5. Reintroduction of T cells from wild-type or *Panx1$^{-/-}$* mice into T cell-deficient Rag1 knockout mice does not affect development of SNI-induced mechanical hypersensitivity. (**A**) Design of T cell adoptive transfer experiments. (SNI image adapted from [55]). (**B**) Representative cytometry plots of CD4 (APC) and CD8 (PE) staining of Thy1.2 positive splenocytes isolated from adoptive transfer mice after SNI. (**C,D**) SNI-induced mechanical hypersensitivity in Rag1KO mice after vehicle treatment (*left*) or after reconstitution with T cells from *Panx1$^{+/+}$* (*middle*) or *Panx1$^{-/-}$* mice (*right*). Statistical analysis of behavioral data as in Fig. 1.

Supplementary Figure 6

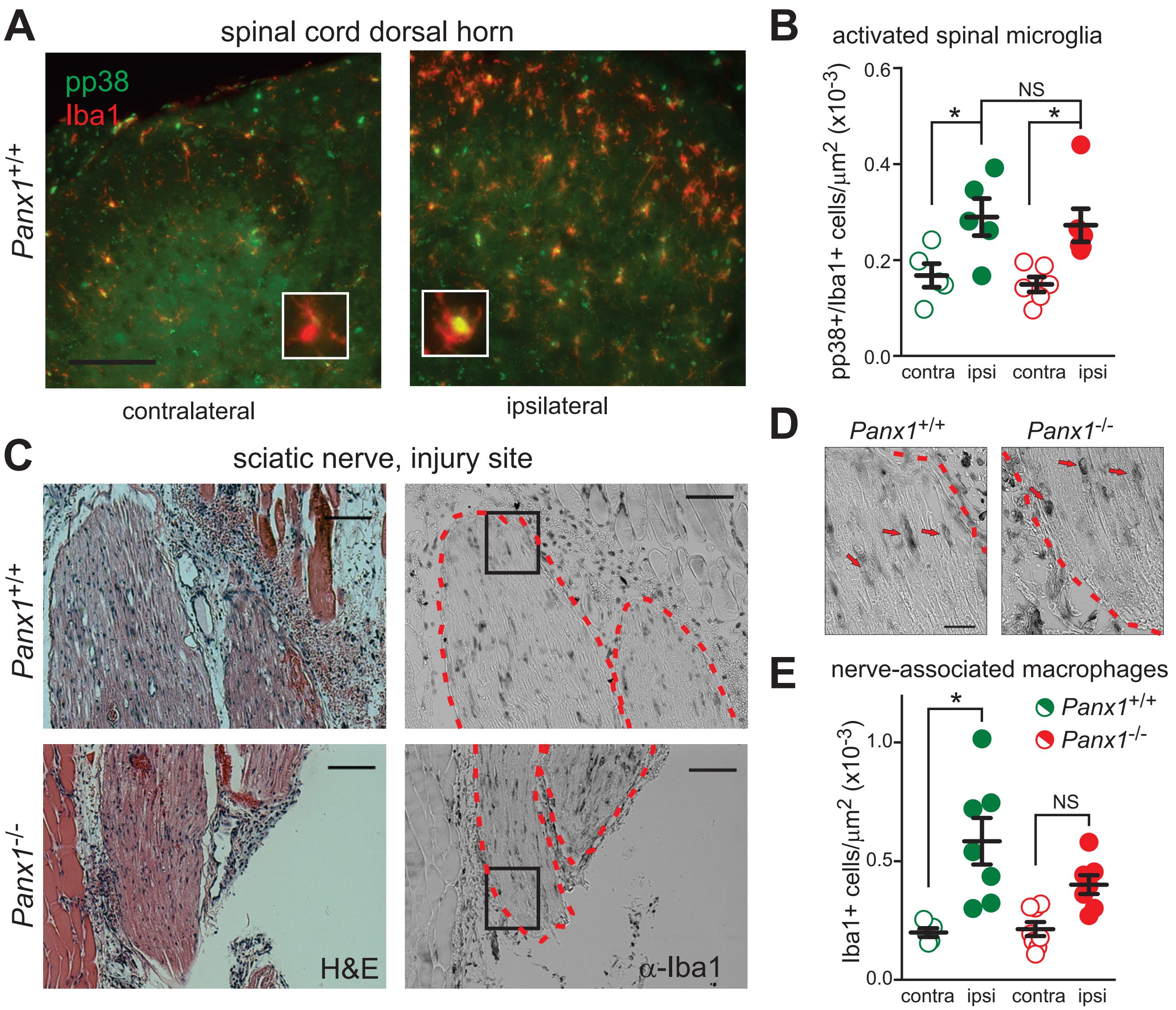

Figure S6. *Panx1$^{-/-}$* mice have lessened peripheral inflammation after nerve injury. (**A**) Example co-staining between Iba1 and pp38 in the dorsal horn of mice after 7 days of injury. Inset on left depicts a typical resting pp38(-) microglia, whereas inset on right shows a pp38(+), activated microglia. Scale bar is 25 μm. (**B**) Quantification of cells with an activated, amoeboid-like Iba1 staining with co-localization of pp38 (*, p<0.05 by two-way ANOVA followed by Sidak's multiple comparisons, n=5-6). (**C**) Example H&E staining of the injured sciatic nerve (left) and Iba1 staining (right). Note the effusion of red blood cells and other cells surrounding the nerve on the H&E stain, which was used to identify the cut end of the nerve. The red border depicts the region within the nerve used for further quantification. Scale bar on the larger images is 100 μm and on the higher magnification image is 25 μm. (**d**) Example high powered images taken from the boxed region in panel (**C**). Arrows are example Iba1(+) cells. (**E**) Quantification of macrophages within the nerve ending. (*, p<0.05 by two-way ANOVA followed by Sidak's multiple comparisons, n=5-8).

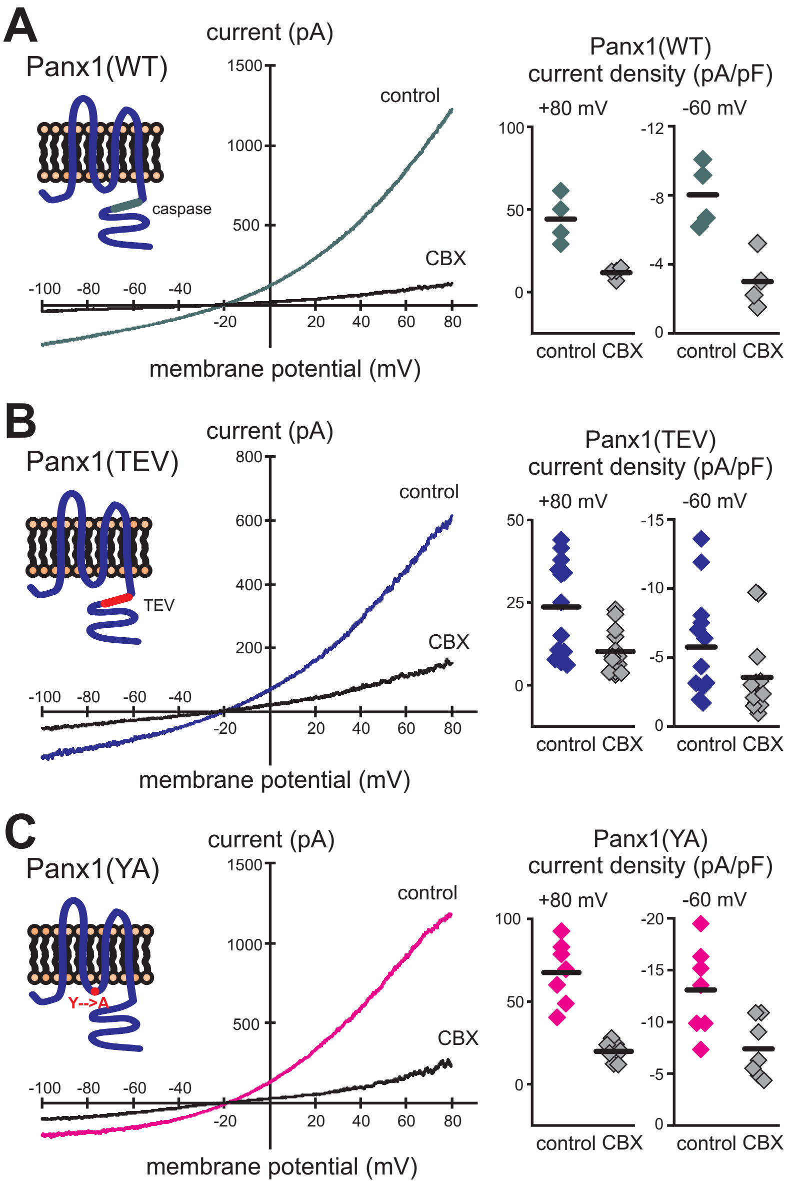

Figure S7. Expression of Panx1 from retroviral vectors in HEK cells. (**A-C**, left) *I*-V curves of Panx1 currents from (**A**) mPanx1(WT), (**B**) mPanx1(TEV), and (**C**) mPanx1(YA) before and after 50 µM CBX application. (**A-C**, right) Summary data of current densities with and without CBX for each vector calculated at +80 mV and at -60 mV. Note that the mouse variant of Panx1 generates basal currents in the absence of activation [28].

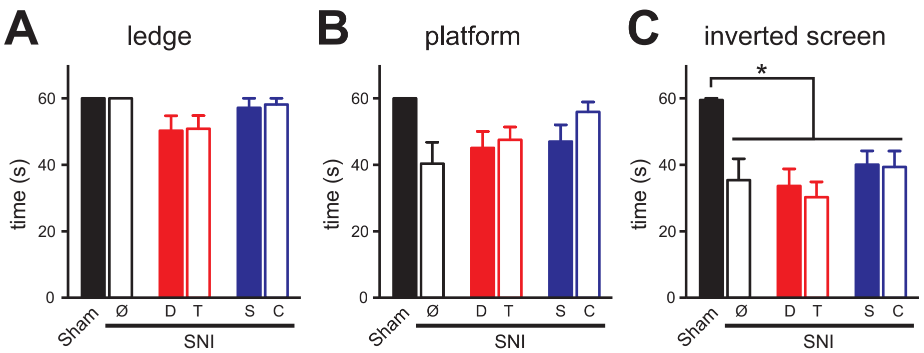

Figure S8. Mice treated daily with Panx1 blockers maintain sensorimotor capabilities. (**A-C**) Sensorimotor function of mice after 7 days of drug (T, trovan or C, CBX) or vehicle treatment (D, DMSO or S, saline). There were no differences in performance between SNI-operated mice receiving no treatment (null symbol) or any of the 7 day drug or vehicle treatments ($p>0.06$, Kruskal-Wallis test). In the last test (**C**, inverted screen), sham mice completed the task better than any of the SNI-operated mice (* $p<0.05$ by Kruskal-Wallis test followed by Dunn's multiple comparisons test).

Table S1: Primers

Name	PrimerBank ID	GenBank Accession	Primer sequence (5’ to 3’)	Amplicon size (bp)
Slc1a3 forward	24233554a1	NM_148938	ACCAAAAGCAACGGAGAAGAG	144
Slc1a3 reverse			GGCATTCCGAAACAGGTAACTC	
Itgam forward	6680484a1	NM_008401	ATGGACGCTGATGGCAATACC	203
Itgam reverse			TCCCCATTCACGTCTCCCA	
Map2 forward	28981317a1	NM_008632	GCCAGCCTCAGAACAAACAG	146
Map2 reverse			AAGGTCTTGGGAGGGAAGAAC	
β-actin forward	6671509a1	NM_007393	GGCTGTATTCCCCTCCATCG	154
β-actin reverse			CCAGTTGGTAACAATGCCATGT	
GAPDH forward	6679937a1	NM_008084	AGGTCGGTGTGAACGGATTTG	123
GAPDH reverse			TGTAGACCATGTAGTTGAGGTCA	
5’-Panx1 homology arm	n/a	n/a	AGGAACCATTCTGCAGGACAGGAA	See Fig. S1B
Common reverse	n/a	n/a	TTATCATAGCAACAGAAATTCTAAGACAGGAATAT	See Fig. S1B
Exon-3 reverse	n/a	n/a	GCTGCAGAGAAGCGCCAGAAGAGTGCG	See Fig. S1B 1
